# Protein serine/threonine phosphatases in tumor microenvironment: a vital player and a promising therapeutic target

**DOI:** 10.7150/thno.104529

**Published:** 2025-01-01

**Authors:** Yiyang Liu, Feng Xia, Chang Zhu, Jia Song, Bufu Tang, Bixiang Zhang, Zhao Huang

**Affiliations:** 1 Hepatic Surgery Center, Tongji Hospital, Tongji Medical College, Huazhong University of Science and Technology, Wuhan, China.; 2 Clinical Medical Research Center of Hepatic Surgery at Hubei Province, Wuhan, China.; 3 Hubei Key Laboratory of Hepato-Pancreatic-Biliary Diseases, Tongji Hospital, Tongji Medical College, Huazhong University of Science and Technology, Wuhan, China.; 4 Key Laboratory of Organ Transplantation, Ministry of Education; Key Laboratory of Organ Transplantation, National Health Commission; Key Laboratory of Organ Transplantation, Chinese Academy of Medical Sciences, Wuhan, China.; 5 Department of Radiation Oncology, Zhongshan Hospital Affiliated to Fudan University, Shanghai, China.

**Keywords:** PSP superfamily, tumor microenvironment, immune therapy, PSPs inhibitors, clinical translation

## Abstract

The tumor microenvironment (TME) is involved in cancer initiation and progression. With advances in the TME field, numerous therapeutic approaches, such as antiangiogenic treatment and immune checkpoint inhibitors, have been inspired and developed. Nevertheless, the sophisticated regulatory effects on the biological balance of the TME remain unclear. Decoding the pathological features of the TME is urgently needed to understand the tumor ecosystem and develop novel antitumor treatments. Protein serine/threonine phosphatases (PSPs) are responsible for inverse protein phosphorylation processes. Aberrant expression and dysfunction of PSPs disturb cellular homeostasis, reprogram metabolic processes and reshape the immune landscape, thereby contributing to cancer progression. Some therapeutic implications, such as the use of PSPs as targets, have drawn the attention of researchers and clinicians. To date, the effects of PSP inhibitors are less satisfactory in real-world practice. With breakthroughs in sequencing technologies, scientists can decipher TME investigations via multiomics and higher resolution. These benefits provide an opportunity to explore the TME in a more comprehensive manner and inspire more findings concerning PSPs in the TME.

The current review starts by introducing the canonical knowledge of PSPs, including their members, structures and posttranslational modifications for activities. We then summarize the functions of PSPs in regulating cellular homeostasis. In particular, we specified the up-to-date roles of PSPs in modulating the immune microenvironment, adopting hypoxia, reprogramming metabolic processes, and responding to extracellular matrix remodeling. Finally, we introduce preclinical PSP inhibitors with translational value and conclude with clinical trials of PSP inhibitors for cancer treatment.

## Introduction

Protein phosphatases, together with protein kinases, mediate reversible and dynamic phosphorylation and dephosphorylation in various biological processes 1. They are classified into protein serine/threonine phosphatase (PSP), protein tyrosine phosphatase (PTP), and haloacid dehalogenase phosphatase (HAD) superfamilies on the basis of the catalytic mechanism. PSPs, such as phosphoprotein phosphatases (PPPs), and PTPs, such as dual-specificity phosphatases (DSPs), dephosphorylate phospho-serine/threonine and phospho-tyrosine residues, respectively. HADs, such as FIIF-associating component of RNA polymerase II C-terminal domain (CTD) phosphatase (FCP), small CTD phosphatase (SCP) and four eyes absent phosphatase, dephosphorylate phospho-serine/threonine and phospho-tyrosine residues 2-4. The PSP superfamily consists of two groups: the PPP and metal-dependent protein phosphatase (PPM) families 2, 4. Studies have revealed the essential roles of PSPs in regulating cell growth, differentiation, signal transduction, and intercellular communication 5-8. Recently, advanced technologies have been applied to decipher the intrinsic crystal structure of PSP complexes. For example, investigations of the PP1-SHOC2-MRAS complex provide a deeper understanding of RAS biology and the RAS/MAPK signaling pathway 9. Furthermore, the selective targeting of PSPs has better inhibitory effects on oncogenic processes 10. Due to their extensive and diverse functions, dysregulated and aberrantly expressed PSP proteins are considered important mediators of the occurrence and development of various diseases, including cancers 11.

The tumor microenvironment (TME) can be traced back to the “seed and soil” concept and has been updated in recent years 12, 13. The TME consists of cellular and acellular components. In fact, the development and fate of tumors depend not only on the intrinsic features of tumor cells but also on the organ where the tumor arises, the stromal cell composition, the extracellular matrix (ECM) and other aspects 14. Jin MZ *et al.* summarized several hallmarks of the TME: hypoxic, acidic and innervated niche, immune, metabolism and mechanical environment 13. Single-cell and spatial transcriptomic data suggest that cancer-TME interactions involve tumor cell evolution and the formation of the tumor ecosystem. For example, tumor cells gain oncogenic mutants, immune evasion ability and organotropism *via* crosstalk with stromal cells 15. Numerous reports have suggested that PSPs are present in the TME and serve as key regulators to modulate hypoxic adaptation, metabolic transition, immune modulation, ECM remodeling, angiogenesis, and intercellular communication 16-20. The substrate specificity and functions of PSPs may result in diverse outcomes in the TME. For example, high levels of PPP1R16B indicate increased immune cell infiltration and improved overall survival (OS) and disease-free survival (DFS) 21. High expression of PPP1R14B contributes to the immunosuppressive TME 22. To obtain a comprehensive understanding of the roles of PSPs in the TME, advanced technologies, such as single-cell transcription analysis, provide new perspectives on their functions, as well as potential drug treatments 23.

In this review, we summarize the roles of PSPs in the TME. Moreover, classical and novel therapeutic approaches are shedding light on the prospects of PSPs in the TME, from the laboratory to the bedside.

## Overview of the PSP superfamily

Seven enzymes are classified as PPPs: PP1, PP2A, PP3/PP2B/calcineurin, PP4, PP5, PP6 and PP7. The sequences identified in the PPPs share a high degree of homology, suggesting that each catalytic subunit may be derived from a common ancestor 24. Previous studies have demonstrated that the majority of PPPs exist as holoenzymes, with catalytic subunits binding to regulatory and/or scaffold subunits 2. PP5 and PP7 exist in monomeric forms and are generally inactive because of their self-inhibiting domains 2, 25. Research on binding proteins with the catalytic subunits of PPPs has established specific domains termed short linear motifs (SLiMs). Several common SLiMs are thought to be involved in phosphatase binding and metal coordination 25. Further investigations revealed that every PPP has specific SLiMs, and the sequence diversity of their amino acids accommodates a range of affinities with various PPP regulators 26.

The majority of PPMs are single-unit phosphatases containing manganese/magnesium ions (Mn^2+^/Mg^2+^) at their activation sites, such as PP2C enzymes. The others are heterodimeric pyruvate dehydrogenase phosphatases (PDPs). Metal ions play a catalytic and central role in the activation of the dephosphorylation reaction for both PPP and PPM. Compared with PPPs, PPMs may rely on additional domains and conserved sequence motifs rather than regulatory subunits to determine substrate specificity. The sequences of the catalytic sites are highly conserved. The PPMs are divided into 12 classes according to their similarity in the sequences of their catalytic domains 25.

Inhibitors, such as okadaic acid, calyculin-A, and tautomycin, can occupy the active sites or SLiMs in PPPs 27. Calcineurin, however, is insensitive to these inhibitors but is vulnerable to cyclosporin (CSP) and FK506 28, 29. For PPMs, specific inhibitors are peptides with sequences that emulate the consensus substrate sequences, such as the AP4-3E peptide, or compounds that bind the specific structure near the catalytic sites, such as GSK2830371 30-32. Additionally, PSPs are regulated by posttranslational modifications (PTMs). Phosphorylation, methyl esterification, protein acetylation, and ubiquitination are the most common PTMs that influence the activity, assembly, expression levels, and other aspects of PSP 2, 33.

## Structures of PSPs

The majority of PPPs function as heterometric dimers with diverse catalytic subunits, regulatory subunits, and/or scaffold subunits. Most PPMs are single-unit enzymes with unique sequences. We use the following well-studied subjects as examples to introduce the structural features of PSPs.

**Figure 1:**
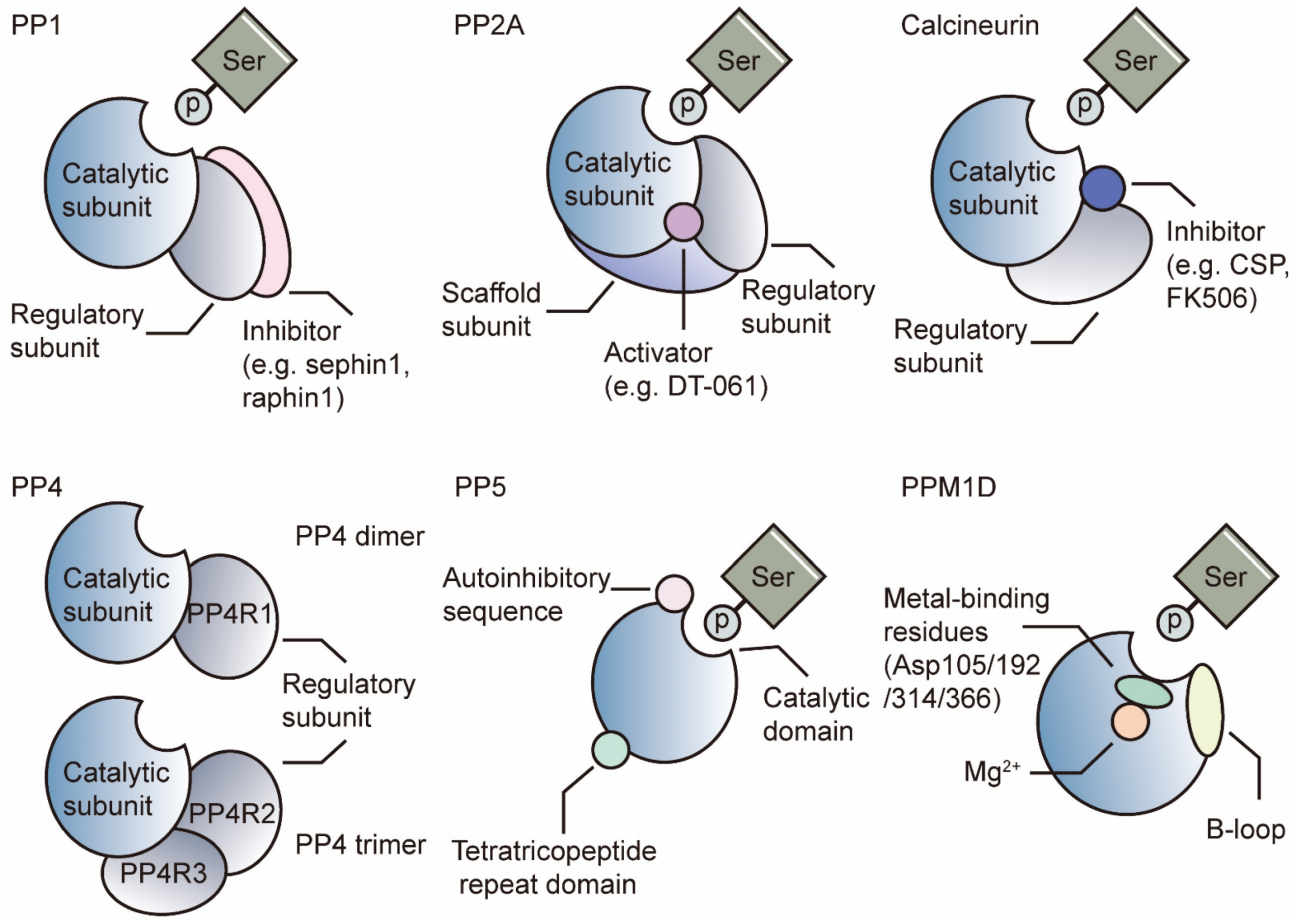
** Structure of the PSPs.** PP1 is comprised of catalytic and regulatory subunits. It can be inhibited by regulatory subunit-specific inhibitors. PP2A is comprised of catalytic, regulatory and scaffold subunits. Activators can stabilize the holoenzyme. Calcineurin is comprised of catalytic and regulatory subunits, which can be inhibited by inhibitors such as CSP and FK506. PP4 can form the dimer with PP4R1 or trimer with PP4R2/PP4R3. PP5 functions as a single subunit enzyme. It features a tetratricopeptide repeat domain and an autoinhibitory sequence. PPM1D is a single-unit enzyme with the B-loop facing the catalytic center. Four Asp residues facilitate the binding of metal iron. Asp: aspartic Acid, CSP: cyclosporin

The PP1 holoenzyme is composed of a catalytic subunit (PP1-C) and one or two regulatory subunit(s) (also known as regulatory interactors of PP1, RIPPOs) (Figure 1) 34, 35. PP1 is encoded by three genes, PPP1CA, PPP1CB and PPP1CC, and consists of three diverse isoforms: PP1A, PP1B, and PP1G. These sequences of PP1-C are quite similar, with several amino acid differences in the N-terminal and C-terminal regions 2, 36. Phosphorylation of PP1 by cyclin A or cyclin B kinases leads to decreased activity *in vitro*, as CDKs can phosphorylate Thr320 in PP1A and Thr311 in PP1G 37. The best-known SLiM is the RVxF motif found in skeletal muscle 38. SLiMs are considered binding sites for distinct RIPPOs. To date, numerous regulatory subunits, such as PUNTS, GADD34/PPP1R15A and MYO18A, have been reported to mediate the specificity of PP1 in various biological processes 7, 39, 40.

PP2A is a heterotrimeric enzyme composed of a catalytic subunit, PP2A-C (encoded by PPP2CA or PPP2CB); a scaffold subunit, PP2A-A (encoded by PPP2R1A or PPP2R1B); and various regulatory subunits, PP2A-B (Figure 1). PP2A-B can be classified into four subfamilies: B55, B56, PR70/72, and striatin (STRN) 41, 42. Like PP1, phosphorylated Tyr307 on the PP2A-C C-terminal tail inhibits the function of PP2A 43. Additionally, reversible carboxymethylation of Leu309 controlled by LCMT-1 and PME-1 regulates holoenzyme assembly 44, 45. The best-known SLiM for PP2A is LSPIxE, which binds B56 for substrate recruitment. Unlike PP1, PP2A-C combines PP2A-A with specific helical repeats rather than SLiMs 25. In addition to postmodifications of PP2A-C, the multiple regulatory subunits direct the specificity of PP2A substrates and may lead to varying outcomes.

Calcineurin comprises an A catalytic subunit and a Ca^2+^-dependent B regulatory subunit (Figure 1). The activation of calcineurin requires Ca^2+^-dependent binding of calmodulin and a subsequent conformational change that displaces the autoinhibitory domain 46. SLiMs are binding sites in catalytic and regulatory subunits for substrate recognition. The well-known substrate of calcineurin is the transcription factor nuclear factor of activated T cells (NFAT), which plays critical roles in T-cell maturation and the normal immune environment 47. The binding site in NFAT has been identified as the PIXIT sequence, which is located in the catalytic A subunit 29. Another SLiM is LxVP, which is mapped at the interface between the catalytic A subunits and regulatory B subunits. The LxVP sequence can be occupied by substrates of calcineurin or two immunosuppressive drugs: CSP and FK506 28, 29.

PP4 complexes consist of a catalytic subunit (PP4-C) and a regulatory subunit (PP4R1) as dimers or PP4R2/PP4R3 as trimers (Figure 1) 48. Like PP2A, PP4 is modified by LCMT-1, which mediates methyl esterification on PP4-C Leu307. Loss of LCMT-1 results in a reduction in the PP4/PP4R1 complex 49.

Unlike those of other PPPs, the catalytic and regulatory domains of PP5 are located on a single peptide 25. PP5 comprises an N-terminal tetratricopeptide repeat (TPR) domain for substrate recognition and a C-terminal PPP catalytic domain 2. The autoinhibitory αJ helix in the C-terminal domain results in low activity of PP5, whereas artificial truncation or depletion of the helix can restore the activity of the catalytic domain (Figure 1) 50, 51. Once HSP90 binds to the TPR, PP5 is activated 52. Subsequent recruitment of Cdc37, a cochaperone, results in the formation of the HSP90-Cdc37-PP5 complex and promotes its combination with various substrates 53.

PPMs function as single subunits with metal ions (Mn^2+^ or Mg^2+^) in the active sites 54. Unlike PPPs, PPMs contain single catalytic subunits (PP2C enzymes) with additional domains and sequences. PP2C enzymes share a highly conserved sequence and are insensitive to PPP inhibitors, such as okadaic acid or microcystin 25. We take PP2Cδ (PPM1D) as an example to introduce the PPMs.

PPM1D is also known as the wild-type p53-induced phosphatase Wip1 because its transcription is induced by P53. PPM1D contains two domains, a carboxy (C)-terminal domain for nuclear translocation signals and a highly conserved amino-terminal phosphatase domain homologous to those of other PP2C enzymes (Figure 1) 55. PPM1D recognizes and dephosphorylates substrates with a diphosphorylated peptide pTXpY sequence, such as MAPK kinases 56. Another sequence, p(S/T)Q, is for substrates, such as ataxia telangiectasia mutated (ATM) kinases 57. PPM1D contains 2 unique inserts in its catalytic domain: the P-loop and B-loop. The P-loop faces the side opposite of the catalytic center, while the B-loop faces the catalytic center 33. The B-loop is the target of some inhibitors, such as GSK2830371 32.

Among PPMs, several amino acid residues have been suggested to ensure the formation and stabilization of the catalytic structure, including Gly61/126/145/198, Glu171, and Arg174/195 in PPM1A 33. In addition, Arg33/186 in PPM1A is essential for substrate recognition 58. Some members, such as PHLPPs, lack the first metal-binding Asp residues (Asp38 of PPM1A), whereas PP2D1 and TAB1 lack the second and third Asp residues (Asp60 or Asp239 of PPM1A), which results in reduced catalytic activity 33.

## Biological function of PSPs

PSPs have multiple functions and participate in various biological and oncogenic processes. Additionally, the regulatory effects vary from combinations with different subunits. In this section, we summarize the results in Table 1 and list several well-studied phosphatases as examples.

PP1 modulates Rb dephosphorylation in the G1/S phase and PTMs of P53 during the cell cycle. In addition, PP1 also functions in cell proliferation and apoptosis, DNA damage, the cellular stress response, glycogen and protein metabolism, and cytoskeleton regulation, among many other processes 5, 11. Multiple combinations of regulatory subunits lead to different signaling pathways and processes. Diverse PP1/RIPPO complexes may have beneficial or detrimental effects on tumors. For example, the PP1-PUNTS complex can prevent the proteasomal degradation of the MYC oncoprotein 59. The PP1-MYO18A-SMAD4 complex dephosphorylates PAK1-Thy423 and inhibits tumor progression in cholangiocarcinoma 7. As a result, the modulation of PP1/RIPPO complexes is considered a feasible and promising approach for tumor treatment 60.

PP2A is involved in many biological processes, including mitosis, the cell cycle, cell proliferation, apoptosis, DNA damage repair (DDR) response, and cell differentiation and development 61, 62. Previous studies have revealed that the PP2A-B55 enzyme can prevent Cyclin B/CDK 1 activation in the G2 phase and stop the G2/M phase transition 63. PP2A-B56 is required for cardiomyocyte maturation and survival, whereas knocking out the B56 regulatory subunit leads to cell apoptosis 64. Additionally, PP2 participates in DDR *via* γ-H2AX dephosphorylation 65.

In tumor cells, the PP2A-B56 and PP2A-B55 complexes are considered antitumor players, whereas the PP2A-STRN3 and PP2A-STRN4 complexes are deemed oncogenic 61, 66, 67. Therefore, interventions on PP2A-B can transform PP2A from being beneficial into an oncoprotein and vice versa. In fact, numerous articles have reported that interventions targeting diverse types of PP2A-B could change the role of PP2A in tumor development, which provides possible and feasible approaches for cancer treatment. Oncoproteins such as cancerous inhibitors of PP2A and SET can inhibit the tumor suppressive effects of the PP2A-B56 complex, whereas cAMP-regulated phosphoprotein 19 interferes with the protective activity of the PP2A-B55 enzyme 61, 66, 67. However, a selective inhibitor of the PP2A-STRN3 complex, SHAP, has antitumor effects on YAP-activated gastric cancers 10. In addition, PP2A regulates Hippo signaling pathway by forming the striatin-interacting phosphatase and kinase (STRIPAK) complex. STRIPAK is a conserved complex containing PP2-C, PP2-A, STRNs, STRN-interacting proteins, sarcolemma membrane-associated protein (SLMAP) and members of the STE20 family of kinases 68. SLMAP recognizes phosphorylated MST1/2, thereby recruiting STRIPAK to dephosphorylate and inactivate MST1/2 and regulating downstream factors of Hippo signaling pathway 69.

The functions of calcineurin, which is a well-known regulator of NFAT, are to mediate T-cell maturation and ensure normal function in benign and malignant diseases 47, 70, 71. Due to their critical role in immune modulation, calcineurin inhibitors have been applied for immunosuppression in various diseases 72. Other PPPs also function in DNA repair, inflammatory modulation, lymphocyte development, tumor growth and survival, and other aspects 2, 73-75.

PPMs participate in vital biological processes, such as cell cycle control, DDR, cell differentiation and immune responses 33. For example, PPM1D mRNA is regulated in a P53-dependent manner. Fibroblasts in PPM1D-deficient mice exhibit a decreased proliferation rate, suggesting an impaired ability to initiate mitosis 76. Furthermore, increased expression of truncated PPM1D also leads to reduction of phosphorylated H2AX, and impairs the P53-mediated G1 cell arrest in breast and colorectal cancer (CRC) 77.

Additionally, PPM1D regulates DDR during tumor development. Phosphorylation of Ser139 in H2AX forms a docking site for subsequent recruitment of other DNA during DDR, while PPM1D disturbs the process by dephosphorylating H2AX 78. Moreover, evidences show that PPM1D is linked with chemo-/radio-resistance. In fact, Miller PG et. al. demonstrates that PPM1D inhibition is a possible approach to enhance chemo-sensitization and response to DDR in P53 wild-type myeloid malignancy 79. Recent study indicates that increased PPM1D protects cells from ionising radiation. In cells with truncated PPM1D, frequent chromosome bridges are observed after radiation exposure. Genome rearrangements protect the cells from radiation-induced cell cycle arrest and senescence and proliferate in the presence of low doses of DNA damage 80.

PPM1D is also required for the optimal functions of T cells and B cells. Mice with PPM1D exhibit increased CD4^+^ T cells and decreased CD8^+^ T cells. The lower proliferative rate of T cells and B cells are detected compared with normal mice. Furthermore, PPM1D knockout mice present skin ulcerations, abnormal lymphoid histopathology and pathogen susceptibility 76.

In addition, widespread participation in tumor immunity, intercellular communication, hypoxic adaptation, and metabolism border the interpretation of PPMs 81-83. The contents will be detailed in the following sections.

## Biological function of other phosphatases

PTPs and HADs are important players to mediate various biological processes in normal cells and tumor cells. In this section, we briefly introduce several typical PTPs and HADs and discuss their biological processes.

PTPs are single-unit enzymes to dephosphorylate Tyr residues, which are divided into Cys-based and His-based families due to the chief catalytic residues involved in nucleophilic substrate attack during catalytic processes 4. Src homology 2 domain-containing inositol polyphosphate 5-phosphatase 2 (SHIP2) is a non-receptor PTP which is ubiquitously expressed and associated with poor prognosis in glioma, melanoma, colon cancer, and breast cancer. Studies have suggested that SHIP2 is a major modulator of PI3K/AKT pathway *via* converting PI(3,4,5)P3 to PI(3,4)P2. Another modulator PTEN converts PI(3,4,5)P3 to PI(4,5)P2. The transduction of PI(3,4,5)P3 to PI(3,4)P2 further triggers downstream targets, such as AKT, mTORC1 and RAF, to ensure cellular survival and proliferation for tumor cells. 84. Furthermore, SHIP2 is reported to interact with c-Cbl ubiquitin ligase to suppress EGRF degradation. 85, 86.

Phosphatase of regenerating liver 3 (PRL3) is a PTP, classified as dual-specificity phosphatase. Studies demonstrate that RPL3 is involved in multiple oncogenic processes, such as activation of EGFR and VEGF pathways 87. PRL3 is demonstrated to reduce IL-4 expression and diminish the inhibitory effects on endothelial cells, thus promoting angiogenesis 88. In addition, PRL3 is demonstrated to mediate lysosomal exocytosis and create an acidic niche to reshape TME 89.

HAD contain a DxDx(V/T) active-site signature motif and work with Asp residue as a catalytic nucleophile and Mg^2+^ as a cofactor 3. The typical HADs are members of FCP and SCP family. The primary substrate of FCP/SCP is the CTD of RNA polymerase II, which contains tandem repeats of a serine-rich heptapeptide 25. FCP1 is the primary phosphatase targeting CTD to dephosphorylate both pSer2 and pSer5 90. Conversely, Scp1 demonstrates minimal catalytic activity towards pSer2, exhibiting a marked preference for pSer5, with a preference ratio of approximately 70 to 1 91.

## Roles of PSPs in the TME

To date, numerous advantages have been identified to illuminate the significance of the TME, from tumor imitation to metastasis outgrowth 14. PSPs widely function in several typical features of the TME to determine the fate of tumors. We introduce the regulation of the TME by PSP in the following two parts: nonimmunological and immunological aspects (Table 2). In nonimmunological aspects, we focus on the interaction of tumors with acellular environment and nonimmune cells. In immunological aspects, we focus on the interaction of tumors with various immune cells.

## Nonimmunological aspect

### Hypoxic adaptation

**Figure 2:**
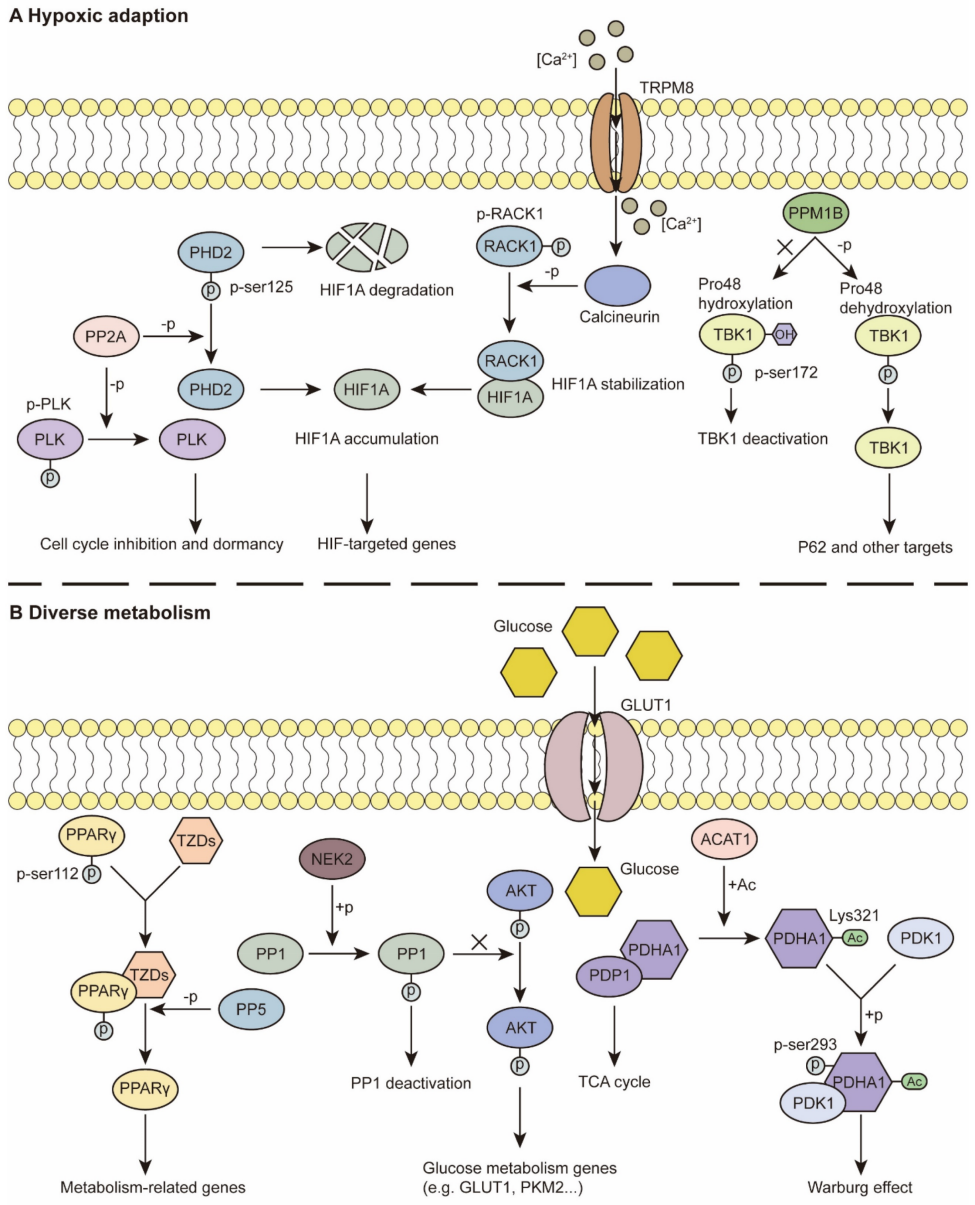
** PSPs in hypoxic adaption and diverse metabolism. (A)** Hypoxic adaption: PP2A dephosphorylates PLK to inhibit cell cycle and promote dormancy. Dephosphorylation of PHD2 at Ser125 leaves HIF1A accumulation. The iron channel TRPM8, acts as a Ca2+ inflow channel, which facilitates the activation of calcineurin. Dephosphorylated RACK1 binds to HIF1A and stabilize HIF1A. Intact HIF1A regulates relative genes which contribute to hypoxic adaption. In normoxia, TBK1 is hydroxylated at Pro48, and can't be dephosphorylated by PPM1B. Under hypoxic conditions, dehydroxylated TBK1 can be dephosphorylated at Ser172, which leads to activation of downstream factors.** (B)** Diverse metabolism: TZDs can bind to PPARγ, which recruits PP5 to dephosphorylate PPARγ at Ser112. Dephosphorylated PPARγ is activated and targets various metabolism-related genes. PP1 is phosphorylated by NEK2. PP1 deactivation facilitates AKT regulation of glucose metabolism genes. PDP1/PDHA1 complex contributes to TCA cycle. Once acetylated on Lys321 by ACAT1, PDHA1 dissociates from PDP1 and binds to PDK1, which leads to Ser293 phosphorylation and the subsequent Warburg effect.

Studies have revealed that hypoxia is a constant feature and that various cancerous properties are involved in hypoxic adaptation. Hypoxia-inducible factors (HIFs) and HIF signaling are classical and essential components of fitness under low-oxygen conditions 13. PSPs have been demonstrated to regulate the expression of HIF1A and downstream effects. For example, the PP2A-B55α complex directly dephosphorylates PHD2 at Ser125, which protects HIF1A from hydroxylation and degradation. Intact HIF1A promoted autophagy-mediated cell survival and colorectal cancer (CRC) development 92 (Figure 2A). In addition, activation of calcineurin by the ion channel TRPM8 leads to RACK1 dephosphorylation and inhibition of its dimerization. This process facilitates the accumulation of HIF1A for the adaptation of prostate cancer cells in the hypoxic niche 93 (Figure 2A). PPM1G negatively regulates the transcriptional activity of HIF1A and promotes its degradation under normoxia and acute hypoxia, thus regulating the hypoxic response 94.

HIF1A can act on PSPs to maintain their cancerous properties. Tiwari A *et al.* reported that HIF1A downregulates the expression of PPP1R1B. Decreased PPP1R1B expression restores the activity of PP1, thus facilitating the protection of P53 from degradation by MDM2 dephosphorylation and regulating invasion and metastasis in pancreatic cancer 16. In ovarian clear cell carcinoma, reduced HIF1A leads to significantly decreased PP2A activity and triggers the Ras pathway 95. PHLPP is a PPM that is negatively regulated by HIF1A. Reduced PHLPP levels contribute to hypoxic adaptation and drug resistance in colon cancer cells 96.

In addition to the HIF-related pathway, other approaches involving hypoxic adaptation involve tumor cells. A few decades ago, a study demonstrated that a hypoxic environment promoted the activity of PP1 toward RB dephosphorylation, thus regulating cell proliferation 97. Later studies revealed that this biological process was due to the reduced expression level of PUNTS, the negative RIPPO 98, 99. In addition, PP1 facilitates fitness in low-oxygen conditions that overexpress GADD34 in ovarian cancer (OC) and is correlated with worse overall survival under hypoxic conditions 100. Previous studies have indicated that PP2A expression levels are increased under hypoxia, whereas hyperoxia hampers the activity of PP2A via posttranslational modification, rather than increasing the abundance of PP2A 101, 102. The overexpression of PP2A has been reported to mediate PLK dephosphorylation, resulting in G1/S phase inhibition and reduced ATP consumption under hypoxia, which contributes to the dormancy and drug resistance of glioblastoma multiforme cells 103 (Figure 2A). The expression level of PP5 is also elevated under hypoxia. The overexpression of PP5 suppresses the hypoxia-induced ASK-1/MKK4/JNK signaling cascade to prevent the apoptotic response 104. In kidney cancer, under normoxia, PPM1B binds to TANK-binding kinase 1 (TBK1) and decreases its activity, whereas under hypoxia, PPM1B cannot dephosphorylate TBK1. TBK1 triggers the downstream Factor P62 and promotes tumorigenesis 105 (Figure 2A).

In addition, SET, an inhibitor regulatory subunit of PP2A, is retained in the cytoplasm of macrophages under hypoxia, thus suppressing the function of PP2A but increasing ERK and P38 signaling, thus regulating the migration and functions of macrophages in the immune response 106.

### Metabolic reprogramming

Cell metabolism contributes to energy consumption by tumor cells and the homeostasis of the TME 13. For example, single-cell sequencing data suggest that PPP2R1A, an LDL-related signature, is involved in lipid metabolism and is associated with worse OS in cutaneous melanoma patients 107. PP5 also plays a tumor-promoting role *via* positive modulation of PPARγ. Once dephosphorylated by PP5, activated PPARγ regulates metabolism-related genes and provides a tumor-promoting TME 108 (Figure 2B). Additionally, PDP2 diminishes lipid peroxidation and inhibits ferroptosis *via* ACSL4 dephosphorylation 109.

For glycogen metabolism, previous reports indicate that inhibition of PP1 activity triggers the AKT/HIF1A pathway and consequent aerobic glycolysis in gastric cancer 110 (Figure 2B). In addition, NSD3 is reported to bind PP1B, leading to STAT3 dephosphorylation, as well as the consequent inhibition of HK2 transcription. This process facilitates glycolysis in lung adenocarcinoma 111. Apart from PP1, more research on glycolysis has focused on PDP1, an activator of pyruvate dehydrogenase (PDH) 83, 112. Fan J *et al.* demonstrated that Lys202 acetylation inhibits PDP1 *via* ACAT1 acetylation, contributing to glycolysis and the Warburg effect in hypoxia 83 (Figure 2B). Moreover, PDP1/PDH histone acetylation has been implicated in glycolysis as well as the consequent radio-resistance of CRC 113.

Recently, PDP1 was reported to play a dual role in maintaining homeostasis. When the expression level of PDP1/PDH is too low under hypoxia, subsequent inhibition of HIF1A transcriptional activity and a reduction in its target gene, pyruvate dehydrogenase kinase 1 (PDK1), partially restore the activity of PDH for cancer cell proliferation. When PDP1/PDH is hyperactivated, the subsequent increase in PDK1 promotes anaerobic glycolysis and lactate production for energy consumption. The intrinsic modulation ensures a stable level of acetyl-CoA, which contributes to adaptation in various TMEs 114. Additionally, in FLT3-ITD-positive acute myeloid leukemia (AML) cells, PDP1 facilitates cellular respiration, even under hypoxic conditions. During reoxygenation, PDP1 promotes the tricarboxylic acid (TCA) cycle for the positive subgroups, which ensures adaptation to variations in oxygen availability in AML bone marrow 17.

### Cancer-associated fibroblasts and ECM remodeling

**Figure 3:**
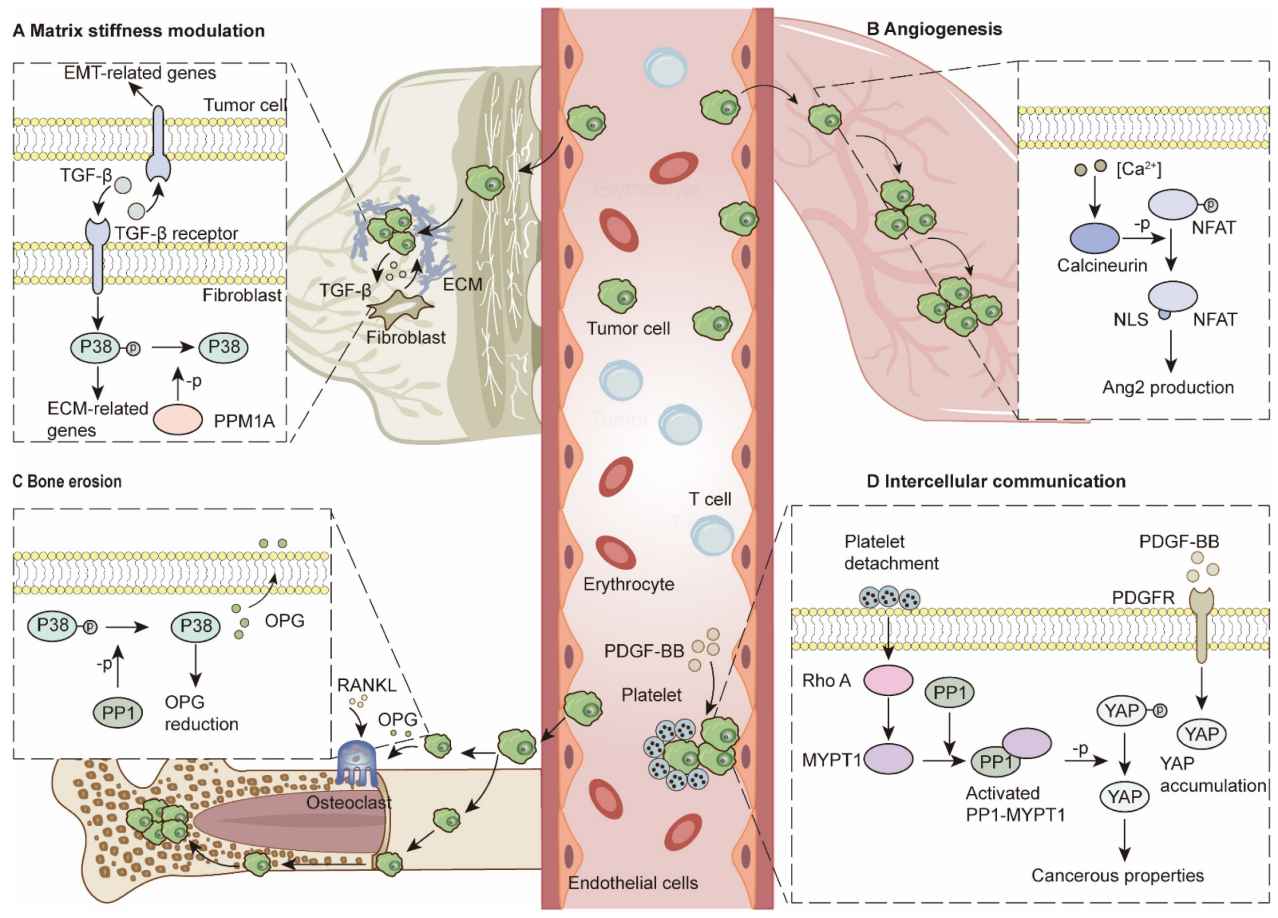
** PSPs in ECM modulation and intercellular communication. (A)** Matrix stiffness modulation. Fibroblasts and tumor cells regulate ECM-related genes and EMT-related genes through TGF-β pathway. PPM1A can dephosphorylate P38 to terminate this process.** (B)** Angiogenesis. Activation of calcineurin/NFAT/Ang2 pathway in endothelial cells contributes to angiogenesis, which provides pre-metastatic niches for lung metastasis.** (C)** Bone erosion. In tumor cells, PP1 dephosphorylates P38 and reduces OPG production. Reduced OPG levels lead to enhanced bone erosion and further bone metastasis.** (D)** Intercellular communication. PDGF-BB induces YAP accumulation. Platelet detachment contributes to PP1-MYPT1 complex activation, which can dephosphorylate YAP for cancerous properties.

Cancer-associated fibroblasts (CAFs) are essential components that modulate stiffness and intercellular crosstalk *via* the secretion of TGF-β, IL-6, and TNF-α, among many other factors 14. For example, fibroblast-to-myofibroblast conversion is reported to nurture the TME and promote migration, invasion and epithelial-to-mesenchymal transition (EMT) in tumor cells *via* TGF-β secretion. Moreover, TGF-β secreted by tumor cells further promotes myofibroblast differentiation, collagen production and ECM stiffness regulation. During this process, PPM1A is the major regulator that can stop it by deactivating the P38/MAPK pathway 82 (Figure 3A). MMPs play important roles in breaking down ECMs 14. PP2A-C upregulation leads to RNA decay of MMPs, whereas inhibition of PP2A can keep MMP2 and MMP9 RNA stable 115. PP5 provides an antitumor environment in various cancers by inhibiting Hsp90α secretion, which synergizes with MMP2 to form an invasive TME 116. In contrast, PP4 acts as a tumor promoter, which is associated with increased MMP gelatinase activity and increased MMP2/MMP9 expression in CRC cells 19.

### Angiogenesis

In addition to CAFs, endothelial cells are also key regulators of the TME. Previously, Martin, M *et al.* reported that PP2A dephosphorylates class IIa HDACs and promotes their nuclear translocation. Downstream factors, such as MMP10, contribute to the formation of vascular-like networks 117. PP2A reportedly modulates ECM stiffness and angiogenesis through VEGF-mediated YAP activation 118. Recent studies have demonstrated the essentiality of PP2A-B55α for endothelial cell survival. PP2A-B55α protects endothelial cells from reactive oxygen species-induced apoptosis by dephosphorylating PHD2 and P38. Vascular network formation provides opportunities for tumor metastasis 119. Depletion of the endogenous calcineurin inhibitor Dscr1 in lung endothelial cells leads to angiogenesis *via* the calcineurin/NFAT/angiopoietin-2 pathway, which provides an optimal premetastatic niche for lung metastasis. Furthermore, excessive VEGF in the TME promotes this process 120 (Figure 3B).

### Stromal cell interactions

In bone marrow, PP1A downregulates osteoprotegerin *via* the P38/MAPK pathway in hepatocellular carcinoma (HCC). Reduced osteoprotegerin (OPG) levels disturb the balance between osteoblasts and osteoclasts, leading to bone resorption and consequent metastasis 20 (Figure 3C). In bone marrow, a previous study demonstrated that differentiated osteoblasts can protect AML cells from SPF-1-induced apoptosis 121. After HDACi treatment, upregulated Nherf1 binds to PP1A to dephosphorylate TAZ in osteoblasts, which inhibits osteoblast-mediated protection against AML 122.

Intercellular communication has been demonstrated to provide tumor cells with cancerous features, increasing their adaptability in various TMEs 14. Recent studies have demonstrated that the PP1‒MYPT1 complex in OC cells can be triggered by dephosphorylation of Thr696 and Thr853 when the complex is incubated with platelets. The functional PP1‒MYPT1 complex in ovarian and colon cancer cells dephosphorylates YAP1 and promotes cancer metastasis 123. Moreover, platelet-derived growth factors (PDGFs) in the TME can activate YAP signaling, whereas PP1 inhibition can counteract this process 124 (Figure 3D). Single-cell RNA sequencing analysis revealed that breast cancer cells can transfer PPP1R1B to tumor endothelial cells *via* extracellular vesicles (EVs), which activate endothelial cells and promote blood vessel formation to alleviate hypoxic conditions 125. In the liver environment, senescent hepatocytes secrete miR-222-5p in exosomes. The microRNA represses its target gene PPP2R2A, leading to decreases in forkhead Box O3, P27 and P21 and accelerating the proliferation and migration abilities of HCC 8.

## Immunity modulation

The TME comprises malignant cells and immune cells, such as T cells, B cells, natural killer (NK) cells, tumor-associated macrophages (TAMs), myeloid-derived suppressor cells (MDSCs), mast cells, granulocytes, dendritic cells (DCs), and other chemokines. The regulation of antitumor immunity is closely associated with tumor development and prognosis 13. Immune checkpoint blockade (ICB) treatments, such as anti-PD1/PDL1 and anti-CTLA4 therapies, which are correlated with immune regulation, showed value in reinvigorating “exhausted” T cells and controlled tumor growth, which have succeeded in some clinical tumor treatments 126. Aberrant expression and dysfunction of PSPs in cancer or immune cells can disturb TME homeostasis, leading to an anti-ICB response and cancer progression.

### Tumor cell-mediated immune response

**Figure 4:**
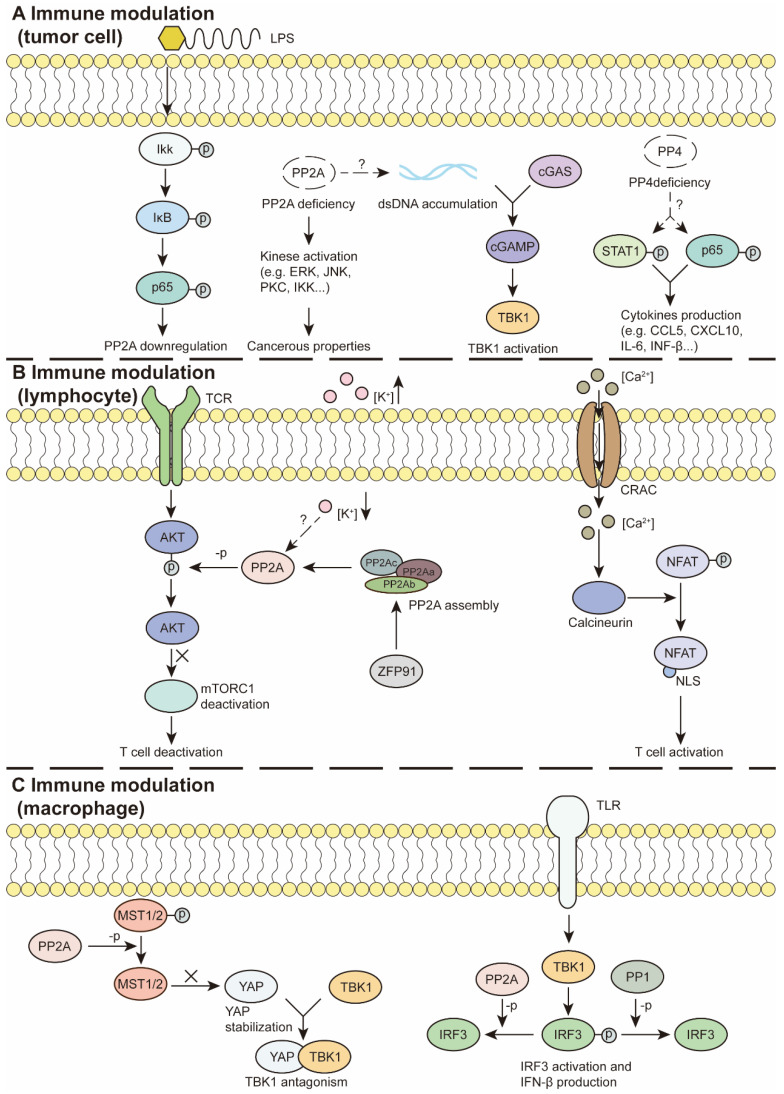
** PSPs in immune modulation. (A)** In tumor cells, inflammatory factors, such as LPS activate NF-κB pathway and reduce PP2A expression. PP2A deficiency promotes kinase activation and acquires cancerous properties. PP2A deficiency activates the GAS-STING pathway to modulate immunity. PP4 deficiency facilitates STAT1 and P65 activation and cytokine production.** (B)** In lymphocytes, PP2A can inhibit TCR-induced mTORC1 activation and T cell activation. Moreover, elevation of the extracellular k^+^ concentration can suppress T cell functions, which is required for intact PP2A. ZFP91 promotes PP2A holoenzyme assembly and downstream effects. Besides, Ca2+ flows through the iron channel CRAC and activates calcineurin, which dephosphorylates NFAT and exposes the NLS. Activated NFAT regulates T cell activation.** (C)** In macrophages, TLR/TBK/IRF3 pathway promotes IFN-β production, whereas PP1 or PP2A can interrupt the process by IRF3 dephosphorylation. Additionally, PP2A dephosphorylates MST1/2 to stabilize YAP, which antagonizes TBK1 and its subsequent effects. LPS: Lipopolysaccharide, NLS: nuclear localization signal, ZFP91: Zinc finger protein 91

The immune response in tumors involves multiple steps, from immune signal activation and chemokine secretion to immune cell recruitment and the antitumor response 126. Indeed, PSPs are demonstrated to regulate signaling pathways and mediate various chemokine productions. Mondal I *et al.* reported that PP2A deficiency contributed to the accumulation of double-stranded DNA and the activation of cGAS-IFN signaling in glioblastoma. Increased IFN production sensitized tumor cells to ICB treatment (Figure 4A) 127. Consistent with these results, the PP2A-RACK1 complex has been demonstrated to dephosphorylate and inactivate TBK1, impeding STING-initiated antitumor immunity in patients with mutant Neurofibromin 2 18. In OC, PP4 inhibition with fostriecin or PPP4C knockdown activates NF-κB and STAT1 pathway. As the results, PP4 inhibition leads to increased secretion of proinflammatory factors, such as CCL5, CXCL10 and IL-6 (Figure 4A). Furthermore, cocultured NK-92 cells with PP4-inhibited OC cells presented significantly elevated IFN-γ production, increased degranulation, and increased NK cell-mediated cytotoxicity. Tumors with PPP4C knockdown exhibit increased infiltration of NK, NK T, CD4^+^ T and CD8^+^ T cells when combined with carboplatin 128. Ubiquitination and degradation of PPM1B trigger NF-κB activation, leading to the secretion of various chemokines, such as CXCL1, CXCL8, and CCL2, and subsequent TAM infiltration, angiogenesis, T-cell dysfunction and resistance to ICB therapy. These results may explain how diverse oncogenic mutations create an immunosuppressive TME in non-small cell lung cancer (NSCLC) 129.

Antigen processing and presentation is a prerequisite for DC-mediated T cell priming and T cell-mediated killing 130. Dissociation of the PP1-GADD34 complex facilitates the cell surface translocation of calreticulin. Exposure to calreticulin is a signal for the recruitment and activation of DCs, CTLs and macrophages, leading to subsequent immunogenic cell death 131. PP2A/PP5 inhibition by LB-100 promotes alternative splicing, which leads to neoantigens presented by MHC-Ⅰ at the surface of colon cancer cells 132.

The inflammatory microenvironment has been implicated in the modulation of PSP activity and subsequent oncogenic processes for tumor cells. Inflammatory stimuli repress the expression level of PP2A *via* NF-κB pathway activation. Reduced PP2A expression in pancreatic cancer cells contributes to ERK, PKC and JNK phosphorylation and promotes cancer development 133. Decreased PP2A expression in NSCLC cells was detected in response to treatment with macrophage culture medium *via* the NF-κB pathway. Furthermore, an activated NF-κB pathway contributes to chemokine production, TAM infiltration, tumor growth and migration in NSCLC 134 (Figure 4A). In addition, inflammation is considered a contributor to changes in intestinal stem cells toward oncogenic phenotypes. This process is associated with PP2A-mediated GSK-3β activation 135.

Evidence from single-cell analysis and clinical data suggests that PSPs are correlated with immune cell infiltration in tumors and patient prognosis. Research suggests that PP2A deficiency in glioblastoma results in enhanced CD8^+^ T cell, DC, and NK cell infiltration and decreased the number of immunosuppressive TAMs 127. In endometrial cancer, high PPP1R16B expression levels indicate the infiltration of B cells, CD4^+^ T cells, CD8^+^ T cells, neutrophils, macrophages and DCs, as well as improved OS and DFS 21. Increased expression of PPP1R14A in gastric cancer is correlated with poor antitumor immunity and poor outcomes 22. High expression levels of PPP1R14B are related to increased MDSC infiltration across cancers 136. In lung adenocarcinoma, PPP1R3G has been suggested to be positively correlated with the infiltration levels of B cells, CD4^+^ T cells and macrophages 137. Some PPM members also indicate tumor outcomes. In CRC, the expression level of PPM1H is negatively correlated with tumor grade. A high level of PP1M1H indicates greater infiltration of M1 macrophages, CD8^+^/CD4^+^ T cells, and activated NK cells and a better survival rate 138.

These inconsistent results may occur within different tumor types. For example, in HCC, high expression of PPM1D is correlated with the expression of inhibitory immune checkpoint markers and worse progression free survival and OS in patients with HCC. In addition, studies have shown that PPM1D contributes to the infiltration of TAMs, Th1 cells, Th2 cells and Tregs 139. Whereas high levels of PPM1D in pancreatic cancer indicate better outcomes 81. Similarly, the expression level of PDP1 in tumors is negatively correlated with CD8^+^ T-cell infiltration, whereas it is positively associated with tumor stage and poor prognosis in invasive breast carcinoma 140. Decreased expression of PDP1 indicates better outcomes in patients with pancreatic adenocarcinoma 81.

The expression levels of some PSPs are correlated with the effects of ICB treatment. The low PPP1R16B expression group had a worse response to anti-PD1/PD-L1 and anti-CTLA4 treatments 21. In pancreatic adenocarcinoma, PPM1K acts as an antitumor agent to negatively regulate the expression level of PD-L1. Decreased expression of PPM1K is associated with reduced infiltration of B cells, mast cells, CD8^+^ cytotoxic T cells, and various CD4^+^ T cells 81.

### Effector T lymphocytes

Previously, PP1 and PP2A were shown to modulate the G0/G1 transition during lymphocyte activation 141. Furthermore, single-cell transcriptome and immune profiling demonstrated that the PP1-GADD34 complex contributes to antitumor immunity. The use of the GADD34 inhibitor Sephin1 is considered to activate the integrated stress response, which leads to decreased CD8^+^ T cells and NK cells and reduced MHC-I, LCK, and SELPLG pathway crosstalk but increased M1 to M2 polarization and immune suppression 23. For PP2A, Zhou P *et al.* demonstrated its participation in T-cell-mediated tumor immunity, as PP2R2D-silenced CD4^+^ and CD8^+^ T cells exhibit increased viability and increased production of interferon-γ (IFN-γ), IL-2 and GM-CSF 6. ZFP91 facilitates the assembly of PP2A, which disturbs mTORC1 activity and restricts glycolysis and T-cell proliferation. The dysfunction of T cells results in an immune-suppressive environment 142 (Figure 4B). In addition, PP2A synergizes with cytotoxic T-lymphocyte-associated protein 4 (CTLA-4) in immune evasion, as CTLA-4 can bind to PP2A for ataxia-telangiectasia mutated autophosphorylation in T-cell apoptosis 143. Furthermore, the contents of the TME also regulate immune activity. An increase in the extracellular potassium concentration due to tumor necrosis leads to PP2A-mediated T-cell suppression (Figure 4B). Inhibition of PP2A with an inhibitor, okadaic acid, or PP2R2D knockdown can restore CD8^+^ T-cell functions 144.

Calcineurin activation dephosphorylates NFAT and induces its nuclear translocation in functional T cells 47 (Figure 4B). The calcineurin-NFAT pathway has been demonstrated to inhibit Treg cell differentiation and improve the immunosuppressive TME 70, 71. Accordingly, targeting calcineurin is considered a possible strategy for treating T-ALL 145. PP4 is required for thymocyte survival and maturation. Depletion of PP4 in T cells leads to aberrant pre-T-cell receptor signaling and can be lethal in mouse embryos 146. Furthermore, PP4R1 reportedly bridges PP4-C to the inhibitor of the NF-κB kinase (IKK) complex, where IKK is dephosphorylated and inactivated. PP4R1 silencing leads to hyperactivation of T cells and malignant properties in cutaneous T-cell lymphoma 147. Additionally, PP4-C reportedly induces apoptosis in leukemic T cells and normal T cells *via* the dephosphorylation of the apoptosis regulator PEA-15 148.

### Regulatory T cells

For Treg cells, Apostolidis SA *et al.* reported that PP2A targeted mTORC1 signaling in a ceramide-dependent manner, which was required for normal functions. Their report suggested that PP2A inhibition in Treg cells may contribute to antitumor immunity 149. Similar results were obtained when cells were treated with nanomedicine containing the immunomodulator dimethylcantharidin (DMC) in mice. DMC serves as a PP2A inhibitor in T cells, which activates mTORC1 signaling, thus promoting cytotoxic T lymphocyte (CTL) infiltration and reducing Treg cell differentiation in the bulk of tumors 150. Moreover, researchers suggest that a multikinase inhibitor, H89, regulates the AKT/PP2A axis, promoting TCR and IL-15 signaling in antitumor immunity 151. The PP2A inhibitor LB-100 enhances chimeric antigen receptor T-cell therapy 152.

### B cells

PP2A is required for the optimal function of normal B cells 153. Inhibiting the mTOR-mediated PP2A/ERK pathway disturbs the proliferation and survival of both normal and malignant B cells 154. Indeed, PP2A affects malignant B cells, and TGF-β-induced apoptosis depends on PP2A activation and subsequent ERK and JNK dephosphorylation 155. In B cells, PP1 activity and actin polymerization contribute to Fc receptor clustering, high affinity for IgG and “inside-out” signaling in neutrophils. This process is speculated to mediate antibody-dependent cellular cytotoxicity and enhance the antitumor response 156.

### In macrophages and MDSCs

An early study revealed that overexpressed PP1 inhibits TLR- or RLR-triggered IFN-β production *via* IRF3 Ser385 and Ser396 dephosphorylation in macrophages. Moreover, the expression level of PP1 is reduced by stimulation with TLR or RLR ligands 157 (Figure 4C). PP2A is another PSP that has been demonstrated to induce macrophage infiltration *via* Rap1-regulated migration, whereas PPP2CA ablation fails to dephosphorylate STAT6, thus prohibiting M1 polarization and TNFα production 158. Inhibition of PP2A by cytoplasmic SET stimulates the P38 and ERK pathways and the expression of M2-related genes. Additionally, SET also regulates the mobility and distribution of tumor-associated macrophages (TAMs) in tumor regions, providing an immunosuppressive environment 106. Previously, PP2A was demonstrated to negatively regulate IFN signaling *via* IRF3 dephosphorylation 159 (Figure 4C). Similar results indicate that the PP2A-STRN4 complex counteracts STING-IFN signaling via the dephosphorylation of the Hippo kinase MST1/2 to maintain the stability of YAP/TAZ in TAMs (Figure 4C). *In vivo* experiments have demonstrated that PP2AC deficiency in macrophages promotes CD8^+^ T-cell infiltration, reduces the number of immunosuppressive TAMs and inhibits tumor growth 160.

The accumulation and activity of MDSCs are increased by the AKT/β-catenin axis, whereas increased PP2A can terminate the pathway and improve the immunosuppressive TME 161. Additionally, inhibition of the PP1-GADD34 complex hampers the production of type I IFN and proinflammatory cytokines in PAMP-activated DCs 162. In addition, the calcium/calcineurin/NFAT axis is required for increased IL-2 production and decreased TGF-β production in DCs. DC-derived polarizing cytokines orchestrate the ratio of Foxp3^+^ Treg cells and IFN-γ^+^ Th1 cells in the TME 163.

## PSP-related therapeutic implications

Given the importance of PSPs in both tumor biology and the TME, from the initial stages to the formation of metastases, a variety of therapeutic strategies have emerged and demonstrated efficacy in both laboratory and clinical experiments (Table 3).

Calcineurin-targeted drugs have been developed and used clinically for decades. The first FDA-approved drug targeting a phosphatase was the calcineurin inhibitor CSP 164. CSP, cyclophilin A, FK506 and pimecrolimus are FDA-approved calcineurin-inhibiting drugs for organ transplantation and atopic dermatitis due to their immunomodulatory functions 165, 166. Coadministration of CSP enhances the effectiveness of dasatinib against chronic myelogenous leukemia (CML) but with low tolerance 167. Recent preclinical studies have demonstrated that rubiginosin B targets calcineurin/NFAT pathway and interrupts Treg cell differentiation. The treatment significantly modulates the immune-suppressive TME and inhibits the growth of CRC cells 70. Zoledronic acid is a bisphosphonate, which indirectly interferes calcineurin-related signaling by regulating gene expression of calcium channel. In patients suffered from breast, bladder, kidney and prostate cancer with skeletal metastases, treatment with zoledronic acid disrupts Ca^2+^/calcineurin/NFAT pathway in Treg cells and represses their immune-suppressive functions 71. However, there are increased risks of carcinogenesis during the usage of calcineurin inhibitors. Inhibiting calcineurin/NFAT pathway increases the expression of ATF3, a negative regulator of P53 transcription, and disrupts P53-induced senescence in tumor cells. In fact, treatment with cyclophilin A or FK506 increases the risk suffering from squamous cell carcinoma 168.

Another promising therapeutic implication is the use of PP2A-targeting drugs. FTY720/fingolimod was originally applied for the treatment of multiple sclerosis because of its immunomodulatory effects. Later studies demonstrated that nonphosphorylated FTY720 indirectly activates PP2A by binding to its inhibitor SET, which presents potential benefits against various neoplasms. Moreover, combined therapy with milatuzumab, gefitinib and dasatinib shows anti-tumor effects in mantle cell lymphoma, triple-negative breast cancer and pancreatic cancer 62. C11 and CM-1231 are analogous to nonphosphorylated FTY720, which has antitumor effects on chronic lymphocytic leukemia (CLL) and AML without immunosuppression or cardiac toxicity 169, 170. Another activator, DT-061 (known as the small-molecule activator of PP2A, SMAP), specifically stabilizes the PP2A-B56α complex by binding to its heterotrimeric regulatory pocket and activates PP2A to dephosphorylate its downstream oncogenic proteins, such as C-MYC 171. Moreover, for those resistant to kinase inhibitors, such as K-RAS-mutant lung cancers, the combination of DT-061 and the MEK inhibitor AZD6244 leads to suppression of the AKT and MYC pathways and attenuation of tumor growth 172. Similarly, for chronic CLL cells with apoptosis resistance caused by impaired Bax/Bak protein activation, DT-061 treatment triggers apoptosis *via* the induction of permeability transition pores in the mitochondria (mPTPs), which increases the expression of cleaved caspase-9 and cleaved PARP, independent of the Bax/Bak pathway 173. In addition, DT-061 activates PP2A and positively regulates GSK3β, which impairs viability in CLL 174.

LB-100, a small molecule inhibitor of PP2A, has been tested in a phase I trial and has shown acceptable safety, tolerability, and antitumor activity in various carcinomas and sarcomas. Stable of the diseases are achieved for patients with atypical carcinoid of the lung, breast cancer, testicular cancer, malignant thymoma, ovarian cancer chondrosarcoma and fibrosarcoma. One patient with pancreatic cancer has a partial response after LB-100 treatment 175. Recent studies have demonstrated that LB-100 sensitizes typical and anaplastic meningioma cells to radiation via DNA repair interruption and G2/M cell cycle arrest 176. LB-100 has been demonstrated to induce chemo-/radio-sensitization in various tumors, such as osteosarcoma, AML, CML and meningioma 177. Additionally, LB-100 reportedly mediates the perturbation of mRNA splicing and sensitizes colon cancer cells to ICB treatments 132. In fact, a phase II trial in CRC patients revealed that the combination of LB-100 and ICB therapy led to complete tumor regression 177. Additionally, preclinical studies revealed that the PP2A inhibitor DMC suppresses Treg cell maturation and promotes CTL infiltration, which alleviates the tumor burden 150. A novel selective inhibitor of the PP2A-STRN3 complex, STRN3-derived Hippo-activating peptide (SHAP), exerts antitumor effects by interrupting MST1/2-mediated YAP activation in gastric cancers. For gastric cancers independent of Hippo pathway, SHAP has limited effects 10.

GSK2830371/GlaxoSmithKline is an inhibitor of PPM1D. Preclinical studies suggest its value in the growth inhibition of hematopoietic tumor cells and PPM1D-amplified breast tumor cells with wild-type P53 32. CCT007093 is another PPM1D inhibitor that induces lethality in ovarian clear cell carcinoma and potently inhibits growth in medulloblastoma 178, 179.

Due to its wide distribution and essential functions in biological processes, PP1 was once deemed “undruggable”. With a deeper investigation of PP1 and other PPPs, some compounds, such as PP2A inhibitors, have shed light on PP1-targeted therapy. More importantly, drugs that target a specific RIPPO are considered feasible and valuable approaches 11. Sephin1, a novel GADD34 inhibitor, impedes PP1-GADD34 complex formation and consequent substrate dephosphorylation and inhibits anaplastic thyroid cancer (ATC) growth. Moreover, PPP1R15A inhibition sensitizes ATC cells to conventional chemical treatment 180. Raphin1 is a PPP1R15B inhibitor. Treating myeloma cells with Raphin1 activates the proapoptotic eIF2α/ATF4/CHOP pathway. Additionally, the use of Raphin1 potentiates the antitumor effect of bortezomib 181.

## Conclusions and prospects

**Figure 5:**
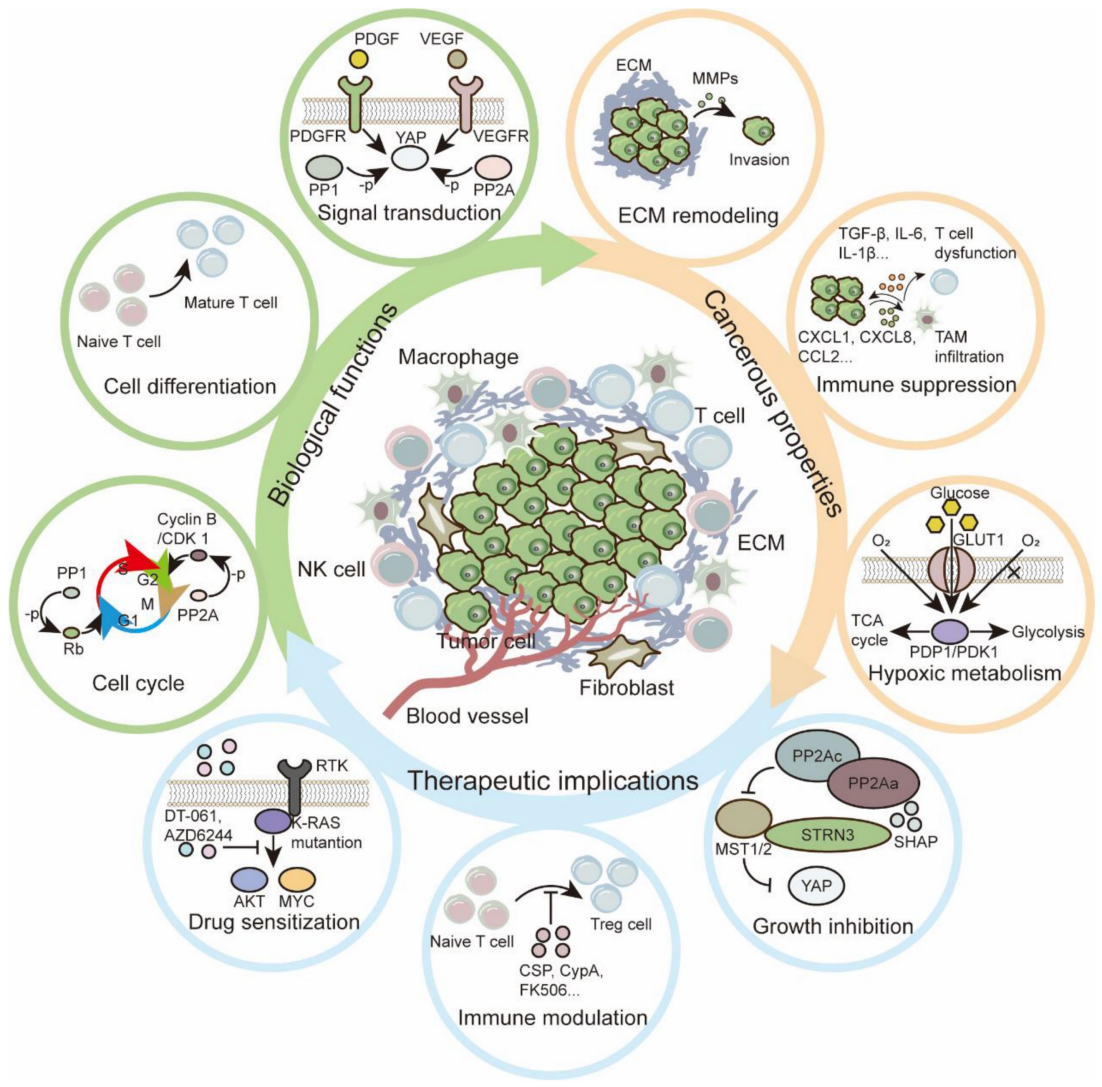
** Functions and therapeutic implications of PSPs in TME.** In TME, PSPs are required for both biological and cancerous functions. Regarding the essential roles of PSPs, both traditional and innovative therapeutic implications are explored for the treatment of tumors.

As major participants, PSPs perform versatile functions *via* phosphorylation and dephosphorylation (Figure 5). With an updated theory of tumor biology, PSPs orchestrate the TME by mediating hypoxic adaptation, immune modulation, diverse metabolism, ECM modifications, and intercellular communication, from initiation to outgrowth of metastasis. Some PSPs are modulated by conserved complexes, such as STRIPAK, which forms a conserve complex to recognize phosphorylated MST1/2 and regulates downstream effectors in signaling transduction. In addition, the functions of PPPs may vary within different regulatory subunits. Considering their beneficial or pathological roles in the TME is arbitrary. Indeed, several FDA-approved drugs have shown promising prospects for targeting PSPs and their subunits; however, several nonnegligible and challenging problems remain unsolved:

First, some PSPs are pivotal players in many biological processes and are widely distributed throughout various cells. Interference with PSPs can be fatal for normal cells. For example, inhibition of the PP2A-B56 complex leads to cardiomyocyte apoptosis 64. The potential side effects of PSP-targeted therapy must be taken into consideration. Second, the functions of some PSP systems require holoenzymes. Diverse combinations of different regulatory subunits may lead to various outcomes 21, 22. As for PP1, PP1-NIPP1 acts as a tumor suppressor for tumor growth inhibition, but also acts as a tumor promotor for migration in cervical cancer 182, 183. Holoenzymes may be beneficial or harmful 60. Unfortunately, the functions of some subunits in tumors still remain unclear. The specific functions of regulatory subunit combinations warrant further investigation. Third, due to the multiple functions of regulatory subunits, drugs are expected to target a specific PSP subunit rather than nonspecific inhibitors 11. Fourth, compensatory effects may occur when phosphatases or kinases are targeted for tumor treatment. In RAS-driven lung cancer, usage of SH2P inhibitors leads to subsequent TGF-β pathway activation, which enhances tumor motility by inducing EMT 184. Different PSP complexes, or specific enzymes with different catalytic subunits and regulatory subunits, display similar functions and phenotypes 185. Whether the oncogenic signaling pathway is rescued by another PSPs, nor a new pathway is triggered when we target a specific PSP? The compensatory effects may be troublesome. Fifth, the development of optimal PSP activators is a difficult problem. Sixth, the prospects of PSPs in cutting-edge fields, such as the tumor-nerve-immunity cycle, warrant further research.

Although several challenges exist in PSP-targeted therapy, targeting biological markers is a possible and accessible approach for drug delivery. Therapeutics modified with tumor marker antibodies have shown promise and mild side effects in the laboratory and clinic 186. Recently, targeting phosphatases with antibody-conjugated nanoparticles successfully delivered drugs to HER2^+^ cancers. Moreover, it makes targeted therapy for “undruggable” disease possible 187.

The wide distribution and pivotal function of PSPs present long-lasting challenges for optimal therapy. With increasing knowledge of the TME, the functions of PSPs will be further elucidated, providing more feasible and valuable drugs for tumor therapy.

## Figures and Tables

**Table 1:** Biological function of PSPs

Enzyme	Type of cell	Function	Ref.
PP1	Various tumor cells	Cell cycle, proliferation	59, 188
	Epithelial cell	Cytoskeleton regulation, cell adhesion	189
	Various tumor cells	Signal transduction	9
	Various tumor cells	DNA damage repair	190
	Ovary cell	Cell stress response	191
	Breast cancer	EMT, invasion	192
	Cholangiocarcinoma	Chemosensitivity	7
PP2A	Cervical carcinoma	Cell cycle, proliferation	10, 63
	Cardiomyocyte	Cell differentiation	64
	Melanocytic cells	senescence	193
	Cervical carcinoma	DNA damage repair	65
	Various tumor cells	Oxidative stress	194
	Gastric cancer	Signal transduction	10
	Colon cancer	Metastasis	195
Calcineurin	T cell	Cell differentiation and maturation	47
	Keratinocyte	Signal transduction	196
	Endothelial cell	Proliferation	197
PP4	Various cells	Cell cycle, DNA damage repair, cell differentiation	75
PP5	Various cells	Cell cycle and growth, DNA damage repair, signal transduction, tumor growth and metastasis	74
PP6	Various cells	Cell cycle and growth, DNA damage repair, signal transduction, cell differentiation, tumor formation and progression	73
PPM1A	Various cells	Cell migration, proliferation, signal transduction	33
PPM1B	Various cells	Necroptosis, senescence, systemic inflammatory response	33
PPM1D	Various cells	Cell cycle regulation, metabolism, cell differentiation, tumor progression	33

**Table 2:** Functions of PSPs in TME

Related properties	Enzyme	Type of cell	Mechanism/pathway	Ref.
Hypoxic adaption	PP1	Pancreatic cancer	PPP1R1B promotes P53 degradation	16
	PP2A	CRC	PP2A-B55α protects HIF1A from hydroxylation and degradation	92
		Glioblastoma multiforme	PP2A mediates cell cycle inhibition and reduced ATP consumption	103
	Calcineurin	Prostate cancer	Calcineurin facilitates accumulation of HIF1A	93
	PP5	Various tumor cells	PP5 suppresses ASK-1/MKK4/JNK pathway	104
	PPM1B	Kidney cancer	PPM1B regulates TBK1 signaling	105
	PPM1G	Renal adenocarcinoma	PPM1G negatively regulates HIF1A expression	94
	PHLPP	Colon cancer	PHLPP is negatively regulated by HIF1A	96
Metabolic reprogramming	PP1	Gastric cancer	PP1 regulates AKT/HIF1A pathway	110
		Lung adenocarcinoma	PP1 inhibits STAT3 signaling	111
	PP5	Bladder cancer	PP5 activates PPARγ signaling	108
	PDP1	Various tumor cells	PDP1 regulates glycolysis and TCA cycle	17, 83, 114
	PDP2	Breast cancer	PDP2 dephosphorylates ACSL4	109
ECM remodeling and angiogenesis	PP2A	Leukemia	PP2A regulates MMPs expression	115
		Endothelial cell	PP2A regulates YAP activation	118
	Calcineurin	Endothelial cell	Calcineurin/NFAT/angiopoietin-2 pathway promotes angiogenesis	120
	PP4	CRC	PP4 is associated with MMPs expression	19
	PP5	Melanoma	PP5 inhibits Hsp90α secretion	116
	PPM1A	Fibroblasts	PPM1A regulates P38/MAPK pathway	82
Stromal cell interactions	PP1	HCC	PP1 regulates P38/MAPK pathway	20
		AML	PP1 regulates osteoblast-mediated protective effects	122
		Various tumor cells	PP1-MYPT1 activates YAP signaling	123, 124
		Breast cancer, endothelial cell	EV-derived PPP1R1B activates endothelial cells	125
	PP2A	HCC	PPP2R2A regulates forkhead box O3, P27 and P21	8
Immune response	PP1	Multiple myeloma	PP1-GADD34 dissociation promotes immunogenic cell death	131
		Various immune cells	PP1-GADD34 mediates integrated stress response	23
		B cell	PP1 regulates to Fc clustering	156
		Macrophage	PP1 inhibits IRF3 signaling	157
		DC	PP1-GADD34 regulates IFN production	162
	PP2A	Various tumor cells	PP2A inhibits STING signaling	18, 127
		Pancreatic cancer, NSCLC	Inflammatory stimuli regulate PP2A expression in tumor cells	133, 134
		CD4^+^/CD8^+^ T cell	PP2A suppresses T cell proliferation, glycolysis and cytokines production	6, 142, 144
		Treg cell	PP2A mediates maturation of Treg cells	149
		B cell	PP2A ensures optimal functions	153
		TAM	SET regulates metabolism, migration, M1 to M2 polarization and STING signaling	106, 158-160
		MDSC	PP2A terminates AKT/β-catenin pathway	161
	Calcineurin	Treg cell	Calcineurin/NFAT pathway regulates Treg differentiation	70, 71
	PP4	DC	Calcineurin/NFAT pathway regulates cytokines production	163
		OC	PP4 regulates inflammatory factors secretion	128
		T cell, lymphoma	PP4-PP4R1 inhibits NF-κB signaling	147
		Normal/leukemic T cell	PP4 dephosphorylates PEA-15	148
	PPM1B	NSCLC	PPM1B regulates NF-κB signaling	129
	PPM1D	HCC	PPM1D is correlated with ICB inhibition	139
	PPM1H	CRC	PPM1G is correlated with immune cell infiltration	138
	PPM1K	Pancreatic adenocarcinoma	PPM1K is correlated with immune cell infiltration	81
		breast invasive carcinoma	PDP1 is negatively correlated with CD8^+^ T cells infiltration	140
	PDP1	Breast invasive carcinoma	PDP1 is negatively correlated with CD8^+^ T cells infiltration	140

**Table 3:** PSP-related therapeutic implications

Compound	Target	Type of cancer	Activator/Inhibitor	Mechanism/Pathway	Comments	Ref.
Sephin1	PP1-GADD34	ATC	Inhibitor	-	Growth inhibition and chemical sensation	180
Raphin1	PP1-PP1R15B	Myeloma	Inhibitor	Activating EIF2α/ATF4/CHOP pathway	Apoptosis promotion and chemical sensation	181
FTY720/ fingolimod	PP2A- B56γ	Lymphoma, breast, pancreatic cancer	Activator	Disrupting PP2A-SET interaction	Growth inhibition in various tumor cells, immunosuppression	62
C11	PP2A- B56γ	CLL	Activator	Disrupting PP2A-SET interaction	Growth inhibition in CLL, analog to FTY720 without immunosuppression	169
CM-1231	PP2A- B56γ	AML	Activator	Disrupting PP2A-SET interaction	Growth inhibition in AML, analog to FTY720 without cardiac toxicity	170
DT-061 /SMAP	PP2A-B56α	CLL, lung cancer	Activator	Inhibiting MEK/mTOR and NOTCH1 pathway, mPTPs-mediated apoptosis	Growth inhibition in K-RAS mutant lung cancer, and CLL	172-174
LB-100	PP2A	Glioblastoma, CRC, lung cancer	Inhibitor	-	Success in phase I and phase II trials, chemo-/radio-sensitization	177
DMC	PP2A	CRC	Inhibitor	Activating PI3K/AKT/mTOR pathway	Immune TME modulation	150
SHAP	PP2A-STRN3	Gastric cancer	Inhibitor	Inhibiting HIPPO pathway	Growth inhibition	10
CSP	Calcineurin	CML	Inhibitor	Inhibiting Wnt/calcineurin/NFAT pathway	Chemo-sensitization, low tolerance	167
Zoledronic acid	Calcineurin	Various cancers with skeletal metastases	Inhibitor	Inhibiting NFAT and IL-2 pathways	Immune TME modulation	71
Rubiginosin B	Calcineurin	CRC	Inhibitor	Inhibiting NFAT pathway	Immune TME modulation	70
GSK2830371/ GlaxoSmithKline	PPM1D	Hematopoietic, breast cancer	Inhibitor	P53 restoration	Growth inhibition	32
CCT007093	PPM1D	Medulloblastoma, OC	Inhibitor	P53 restoration	Growth inhibition and induced lethality	178, 179

## Abbreviations

AML: acute myeloid leukemia
ATC: anaplastic thyroid cancer
CAF: cancer-associated fibroblast
CLL: chronic lymphocytic leukemia
CML: chronic myeloid leukemia
CRC: colorectal cancer
CSP: cyclosporin
CTD: C-terminal domain
CTL: cytotoxic T cell
CTLA-4: cytotoxic T-lymphocyte-associated protein 4
DDR: DNA damage repair
DFS: disease free survival
DMC: demethylcantharidin
DSP: dual-specificity phosphatase
ECM: extracellular matrix
EMT: epithelial to mesenchymal transition
EV: extracellular vesicle
FCP: FIIF-associating component of RNA polymerase II CTD phosphatase
HCC: hepatocellular carcinoma
HIF: Hypoxia-inducible factor
ICB: immune checkpoint blockade
IFN: interferon
IKK: inhibitor of NF-κB kinase
MDSC: myeloid-derived suppressor cell
mPTPs: permeability transition pores in the mitochondria
NFAT: nuclear factor of activated T cells
NSCLC: non-small cell lung cancer
OC: ovarian cancer
OPG: osteoprotegerin
OS: overall survival
PDGF: platelet-derived growth factor
PDH: pyruvate dehydrogenase
PDK1: pyruvate dehydrogenase kinase 1
PDP: pyruvate dehydrogenase phosphatase
PPM: metal-dependent protein phosphatase
PPP: phosphoprotein phosphatase
PRL3: phosphatase of regenerating liver 3
PSP: protein serine/threonine phosphatase
PTM: post translational modification
SCP: small CTD phosphatase
SHIP2: Src homology 2 domain-containing inositol polyphosphate 5-phosphatase 2
SLiM: short linear motif
SLMAP: sarcolemma membrane-associated protein
STRIPAK: striatin-interacting phosphatase and kinase
RIPPO: regulatory interactor of PP1
SMAP: small-molecule activator of PP2A
STRN: striatin
TAM: tumor-associated macrophage
TBK1: TANK Binding Kinase 1
TCA: tricarboxylic acid
TME: tumor microenvironment
TPR: tetratricopeptide repeat

## References

[1] Brautigan DL (1995). Flicking the switches: phosphorylation of serine/threonine protein phosphatases. Semin Cancer Biol.

[2] Brautigan DL, Shenolikar S (2018). Protein Serine/Threonine Phosphatases: Keys to Unlocking Regulators and Substrates. Annu Rev Biochem.

[3] Seifried A, Schultz J, Gohla A (2013). Human HAD phosphatases: structure, mechanism, and roles in health and disease. FEBS J.

[4] Stanford SM, Bottini N (2023). Targeting protein phosphatases in cancer immunotherapy and autoimmune disorders. Nat Rev Drug Discov.

[5] Ceulemans H, Bollen M (2004). Functional diversity of protein phosphatase-1, a cellular economizer and reset button. Physiol Rev.

[6] Zhou P, Shaffer DR, Alvarez Arias DA, Nakazaki Y, Pos W, Torres AJ (2014). *In vivo* discovery of immunotherapy targets in the tumour microenvironment. Nature.

[7] Liu J, Ren G, Li K, Liu Z, Wang Y, Chen T (2022). The Smad4-MYO18A-PP1A complex regulates beta-catenin phosphorylation and pemigatinib resistance by inhibiting PAK1 in cholangiocarcinoma. Cell Death Differ.

[8] Ma Y, Li S, Ye S, Luo S, Wei L, Su Y (2024). The role of miR-222-2p in exosomes secreted by hexavalent chromium-induced premature senescent hepatocytes as a SASP component. Environ Pollut.

[9] Hauseman ZJ, Fodor M, Dhembi A, Viscomi J, Egli D, Bleu M (2022). Structure of the MRAS-SHOC2-PP1C phosphatase complex. Nature.

[10] Tang Y, Fang G, Guo F, Zhang H, Chen X, An L (2020). Selective Inhibition of STRN3-Containing PP2A Phosphatase Restores Hippo Tumor-Suppressor Activity in Gastric Cancer. Cancer Cell.

[11] Felgueiras J, Jeronimo C, Fardilha M (2020). Protein phosphatase 1 in tumorigenesis: is it worth a closer look?. Biochim Biophys Acta Rev Cancer.

[12] Paget S (1989). The distribution of secondary growths in cancer of the breast. 1889. Cancer Metastasis Rev.

[13] Jin MZ, Jin WL (2020). The updated landscape of tumor microenvironment and drug repurposing. Signal Transduct Target Ther.

[14] de Visser KE, Joyce JA (2023). The evolving tumor microenvironment: From cancer initiation to metastatic outgrowth. Cancer Cell.

[15] Seferbekova Z, Lomakin A, Yates LR, Gerstung M (2023). Spatial biology of cancer evolution. Nat Rev Genet.

[16] Tiwari A, Tashiro K, Dixit A, Soni A, Vogel K, Hall B (2020). Loss of HIF1A From Pancreatic Cancer Cells Increases Expression of PPP1R1B and Degradation of p53 to Promote Invasion and Metastasis. Gastroenterology.

[17] Alshamleh I, Kurrle N, Makowka P, Bhayadia R, Kumar R, Susser S (2023). PDP1 is a key metabolic gatekeeper and modulator of drug resistance in FLT3-ITD-positive acute myeloid leukemia. Leukemia.

[18] Meng F, Yu Z, Zhang D, Chen S, Guan H, Zhou R (2021). Induced phase separation of mutant NF2 imprisons the cGAS-STING machinery to abrogate antitumor immunity. Mol Cell.

[19] Li X, Liang L, Huang L, Ma X, Li D, Cai S (2015). High expression of protein phosphatase 4 is associated with the aggressive malignant behavior of colorectal carcinoma. Mol Cancer.

[20] Huang Z, Chu L, Liang J, Tan X, Wang Y, Wen J (2021). H19 Promotes HCC Bone Metastasis Through Reducing Osteoprotegerin Expression in a Protein Phosphatase 1 Catalytic Subunit Alpha/p38 Mitogen-Activated Protein Kinase-Dependent Manner and Sponging microRNA 200b-3p. Hepatology.

[21] Liu X, Feng D, Wang W, Liang J, Yu H, Ling B (2023). Tumor Microenvironment CD8 T and Treg Cells-related Genes Signature Distinguishes Distinct Prognosis and Targeted Therapies Response in Endometrial Cancer. J Immunother.

[22] Liu Y, Wu J, Huang W, Weng S, Wang B, Chen Y (2020). Development and validation of a hypoxia-immune-based microenvironment gene signature for risk stratification in gastric cancer. J Transl Med.

[23] Wang R, Zhang Y, Guo S, Pei S, Guo W, Wu Z (2023). Single-cell RNA sequencing reveals the suppressive effect of PPP1R15A inhibitor Sephin1 in antitumor immunity. iScience.

[24] Vincent JB, Averill BA (1990). Sequence homology between purple acid phosphatases and phosphoprotein phosphatases. Are phosphoprotein phosphatases metalloproteins containing oxide-bridged dinuclear metal centers?. FEBS Lett.

[25] Shi Y (2009). Serine/threonine phosphatases: mechanism through structure. Cell.

[26] Bollen M, Peti W, Ragusa MJ, Beullens M (2010). The extended PP1 toolkit: designed to create specificity. Trends Biochem Sci.

[27] Favre B, Turowski P, Hemmings BA (1997). Differential inhibition and posttranslational modification of protein phosphatase 1 and 2A in MCF7 cells treated with calyculin-A, okadaic acid, and tautomycin. J Biol Chem.

[28] Huai Q, Kim HY, Liu Y, Zhao Y, Mondragon A, Liu JO (2002). Crystal structure of calcineurin-cyclophilin-cyclosporin shows common but distinct recognition of immunophilin-drug complexes. Proc Natl Acad Sci U S A.

[29] Aramburu J, Yaffe MB, Lopez-Rodriguez C, Cantley LC, Hogan PG, Rao A (1999). Affinity-driven peptide selection of an NFAT inhibitor more selective than cyclosporin A. Science.

[30] Chuman Y, Yagi H, Fukuda T, Nomura T, Matsukizono M, Shimohigashi Y (2008). Characterization of the active site and a unique uncompetitive inhibitor of the PPM1-type protein phosphatase PPM1D. Protein Pept Lett.

[31] Hayashi R, Tanoue K, Durell SR, Chatterjee DK, Jenkins LM, Appella DH (2011). Optimization of a cyclic peptide inhibitor of Ser/Thr phosphatase PPM1D (Wip1). Biochemistry.

[32] Gilmartin AG, Faitg TH, Richter M, Groy A, Seefeld MA, Darcy MG (2014). Allosteric Wip1 phosphatase inhibition through flap-subdomain interaction. Nat Chem Biol.

[33] Kamada R, Kudoh F, Ito S, Tani I, Janairo JIB, Omichinski JG (2020). Metal-dependent Ser/Thr protein phosphatase PPM family: Evolution, structures, diseases and inhibitors. Pharmacol Ther.

[34] Terry-Lorenzo RT, Elliot E, Weiser DC, Prickett TD, Brautigan DL, Shenolikar S (2002). Neurabins recruit protein phosphatase-1 and inhibitor-2 to the actin cytoskeleton. J Biol Chem.

[35] Dancheck B, Ragusa MJ, Allaire M, Nairn AC, Page R, Peti W (2011). Molecular investigations of the structure and function of the protein phosphatase 1-spinophilin-inhibitor 2 heterotrimeric complex. Biochemistry.

[36] Korrodi-Gregorio L, Esteves SL, Fardilha M (2014). Protein phosphatase 1 catalytic isoforms: specificity toward interacting proteins. Transl Res.

[37] Dohadwala M, da Cruz e Silva EF, Hall FL, Williams RT, Carbonaro-Hall DA, Nairn AC (1994). Phosphorylation and inactivation of protein phosphatase 1 by cyclin-dependent kinases. Proc Natl Acad Sci U S A.

[38] Wu J, Liu J, Thompson I, Oliver CJ, Shenolikar S, Brautigan DL (1998). A conserved domain for glycogen binding in protein phosphatase-1 targeting subunits. FEBS Lett.

[39] Wei Y, Redel C, Ahlner A, Lemak A, Johansson-Akhe I, Houliston S (2022). The MYC oncoprotein directly interacts with its chromatin cofactor PNUTS to recruit PP1 phosphatase. Nucleic Acids Res.

[40] Shi W, Sun C, He B, Xiong W, Shi X, Yao D (2004). GADD34-PP1c recruited by Smad7 dephosphorylates TGFbeta type I receptor. J Cell Biol.

[41] Cho US, Xu W (2007). Crystal structure of a protein phosphatase 2A heterotrimeric holoenzyme. Nature.

[42] Xu Y, Chen Y, Zhang P, Jeffrey PD, Shi Y (2008). Structure of a protein phosphatase 2A holoenzyme: insights into B55-mediated Tau dephosphorylation. Mol Cell.

[43] Chen J, Parsons S, Brautigan DL (1994). Tyrosine phosphorylation of protein phosphatase 2A in response to growth stimulation and v-src transformation of fibroblasts. J Biol Chem.

[44] Stanevich V, Jiang L, Satyshur KA, Li Y, Jeffrey PD, Li Z (2011). The structural basis for tight control of PP2A methylation and function by LCMT-1. Mol Cell.

[45] Xing Y, Li Z, Chen Y, Stock JB, Jeffrey PD, Shi Y (2008). Structural mechanism of demethylation and inactivation of protein phosphatase 2A. Cell.

[46] Li H, Rao A, Hogan PG (2011). Interaction of calcineurin with substrates and targeting proteins. Trends Cell Biol.

[47] Macian F (2005). NFAT proteins: key regulators of T-cell development and function. Nat Rev Immunol.

[48] Chowdhury D, Xu X, Zhong X, Ahmed F, Zhong J, Liao J (2008). A PP4-phosphatase complex dephosphorylates gamma-H2AX generated during DNA replication. Mol Cell.

[49] Hwang J, Lee JA, Pallas DC (2016). Leucine Carboxyl Methyltransferase 1 (LCMT-1) Methylates Protein Phosphatase 4 (PP4) and Protein Phosphatase 6 (PP6) and Differentially Regulates the Stable Formation of Different PP4 Holoenzymes. J Biol Chem.

[50] Kang H, Sayner SL, Gross KL, Russell LC, Chinkers M (2001). Identification of amino acids in the tetratricopeptide repeat and C-terminal domains of protein phosphatase 5 involved in autoinhibition and lipid activation. Biochemistry.

[51] Chen MX, Cohen PT (1997). Activation of protein phosphatase 5 by limited proteolysis or the binding of polyunsaturated fatty acids to the TPR domain. FEBS Lett.

[52] Ramsey AJ, Chinkers M (2002). Identification of potential physiological activators of protein phosphatase 5. Biochemistry.

[53] Oberoi J, Guiu XA, Outwin EA, Schellenberger P, Roumeliotis TI, Choudhary JS (2022). HSP90-CDC37-PP5 forms a structural platform for kinase dephosphorylation. Nat Commun.

[54] Das AK, Helps NR, Cohen PT, Barford D (1996). Crystal structure of the protein serine/threonine phosphatase 2C at 2.0 A resolution. EMBO J.

[55] Nahta R, Castellino RC (2021). Phosphatase magnesium-dependent 1 delta (PPM1D), serine/threonine protein phosphatase and novel pharmacological target in cancer. Biochem Pharmacol.

[56] Yamaguchi H, Minopoli G, Demidov ON, Chatterjee DK, Anderson CW, Durell SR (2005). Substrate specificity of the human protein phosphatase 2Cdelta, Wip1. Biochemistry.

[57] Yamaguchi H, Durell SR, Chatterjee DK, Anderson CW, Appella E (2007). The Wip1 phosphatase PPM1D dephosphorylates SQ/TQ motifs in checkpoint substrates phosphorylated by PI3K-like kinases. Biochemistry.

[58] Li R, Gong Z, Pan C, Xie DD, Tang JY, Cui M (2013). Metal-dependent protein phosphatase 1A functions as an extracellular signal-regulated kinase phosphatase. FEBS J.

[59] Dingar D, Tu WB, Resetca D, Lourenco C, Tamachi A, De Melo J (2018). MYC dephosphorylation by the PP1/PNUTS phosphatase complex regulates chromatin binding and protein stability. Nat Commun.

[60] Matos B, Howl J, Jeronimo C, Fardilha M (2021). Modulation of serine/threonine-protein phosphatase 1 (PP1) complexes: A promising approach in cancer treatment. Drug Discov Today.

[61] Fowle H, Zhao Z, Grana X (2019). PP2A holoenzymes, substrate specificity driving cellular functions and deregulation in cancer. Adv Cancer Res.

[62] Mazhar S, Taylor SE, Sangodkar J, Narla G (2019). Targeting PP2A in cancer: Combination therapies. Biochim Biophys Acta Mol Cell Res.

[63] Burgess A, Vigneron S, Brioudes E, Labbe JC, Lorca T, Castro A (2010). Loss of human Greatwall results in G2 arrest and multiple mitotic defects due to deregulation of the cyclin B-Cdc2/PP2A balance. Proc Natl Acad Sci U S A.

[64] Varadkar P, Despres D, Kraman M, Lozier J, Phadke A, Nagaraju K (2014). The protein phosphatase 2A B56gamma regulatory subunit is required for heart development. Dev Dyn.

[65] Chowdhury D, Keogh MC, Ishii H, Peterson CL, Buratowski S, Lieberman J (2005). gamma-H2AX dephosphorylation by protein phosphatase 2A facilitates DNA double-strand break repair. Mol Cell.

[66] Kauko O, Westermarck J (2018). Non-genomic mechanisms of protein phosphatase 2A (PP2A) regulation in cancer. Int J Biochem Cell Biol.

[67] Haanen TJ 3rd, O'Connor CM, Narla G (2022). Biased holoenzyme assembly of protein phosphatase 2A (PP2A): From cancer to small molecules. J Biol Chem.

[68] Goudreault M, D'Ambrosio LM, Kean MJ, Mullin MJ, Larsen BG, Sanchez A (2009). A PP2A phosphatase high density interaction network identifies a novel striatin-interacting phosphatase and kinase complex linked to the cerebral cavernous malformation 3 (CCM3) protein. Mol Cell Proteomics.

[69] Zheng Y, Liu B, Wang L, Lei H, Pulgar Prieto KD, Pan D (2017). Homeostatic Control of Hpo/MST Kinase Activity through Autophosphorylation-Dependent Recruitment of the STRIPAK PP2A Phosphatase Complex. Cell Rep.

[70] Geng CA, Chen FY, Zheng JB, Liao P, Li TZ, Zhang XM (2023). Rubiginosin B selectively inhibits Treg cell differentiation and enhances anti-tumor immune responses by targeting calcineurin-NFAT signaling pathway. Phytomedicine.

[71] Sarhan D, Leijonhufvud C, Murray S, Witt K, Seitz C, Wallerius M (2017). Zoledronic acid inhibits NFAT and IL-2 signaling pathways in regulatory T cells and diminishes their suppressive function in patients with metastatic cancer. Oncoimmunology.

[72] Roy J, Cyert MS (2020). Identifying New Substrates and Functions for an Old Enzyme: Calcineurin. Cold Spring Harb Perspect Biol.

[73] Ohama T (2019). The multiple functions of protein phosphatase 6. Biochim Biophys Acta Mol Cell Res.

[74] Sager RA, Dushukyan N, Woodford M, Mollapour M (2020). Structure and function of the co-chaperone protein phosphatase 5 in cancer. Cell Stress Chaperones.

[75] Park J, Lee DH (2020). Functional roles of protein phosphatase 4 in multiple aspects of cellular physiology: a friend and a foe. BMB Rep.

[76] Choi J, Nannenga B, Demidov ON, Bulavin DV, Cooney A, Brayton C (2002). Mice deficient for the wild-type p53-induced phosphatase gene (Wip1) exhibit defects in reproductive organs, immune function, and cell cycle control. Mol Cell Biol.

[77] Kleiblova P, Shaltiel IA, Benada J, Sevcik J, Pechackova S, Pohlreich P (2013). Gain-of-function mutations of PPM1D/Wip1 impair the p53-dependent G1 checkpoint. J Cell Biol.

[78] Moon SH, Nguyen TA, Darlington Y, Lu X, Donehower LA (2010). Dephosphorylation of gamma-H2AX by WIP1: an important homeostatic regulatory event in DNA repair and cell cycle control. Cell Cycle.

[79] Miller PG, Sperling AS, Mayerhofer C, McConkey ME, Ellegast JM, Da Silva C (2023). PPM1D modulates hematopoietic cell fitness and response to DNA damage and is a therapeutic target in myeloid malignancy. Blood.

[80] Stoyanov M, Martinikova AS, Matejkova K, Horackova K, Zemankova P, Burdova K (2024). PPM1D activity promotes cellular transformation by preventing senescence and cell death. Oncogene.

[81] Zhuang Y, Lan S, Zhong W, Huang F, Peng J, Zhang S (2023). Comprehensive Analysis of PPMs in Pancreatic Adenocarcinoma Indicates the Value of PPM1K in the Tumor Microenvironment. Cancers (Basel).

[82] Shukla A, Edwards R, Yang Y, Hahn A, Folkers K, Ding J (2014). CLIC4 regulates TGF-beta-dependent myofibroblast differentiation to produce a cancer stroma. Oncogene.

[83] Fan J, Shan C, Kang HB, Elf S, Xie J, Tucker M (2014). Tyr phosphorylation of PDP1 toggles recruitment between ACAT1 and SIRT3 to regulate the pyruvate dehydrogenase complex. Mol Cell.

[84] Pedicone C, Meyer ST, Chisholm JD, Kerr WG (2021). Targeting SHIP1 and SHIP2 in Cancer. Cancers (Basel).

[85] Prasad NK, Decker SJ (2005). SH2-containing 5'-inositol phosphatase, SHIP2, regulates cytoskeleton organization and ligand-dependent down-regulation of the epidermal growth factor receptor. J Biol Chem.

[86] Prasad NK, Tandon M, Badve S, Snyder PW, Nakshatri H (2008). Phosphoinositol phosphatase SHIP2 promotes cancer development and metastasis coupled with alterations in EGF receptor turnover. Carcinogenesis.

[87] Chia PL, Ang KH, Thura M, Zeng Q (2023). PRL3 as a therapeutic target for novel cancer immunotherapy in multiple cancer types. Theranostics.

[88] Guo K, Li J, Wang H, Osato M, Tang JP, Quah SY (2006). PRL-3 initiates tumor angiogenesis by recruiting endothelial cells *in vitro* and *in vivo*. Cancer Res.

[89] Funato Y, Yoshida A, Hirata Y, Hashizume O, Yamazaki D, Miki H (2020). The Oncogenic PRL Protein Causes Acid Addiction of Cells by Stimulating Lysosomal Exocytosis. Dev Cell.

[90] Hausmann S, Erdjument-Bromage H, Shuman S (2004). Schizosaccharomyces pombe carboxyl-terminal domain (CTD) phosphatase Fcp1: distributive mechanism, minimal CTD substrate, and active site mapping. J Biol Chem.

[91] Zhang Y, Kim Y, Genoud N, Gao J, Kelly JW, Pfaff SL (2006). Determinants for dephosphorylation of the RNA polymerase II C-terminal domain by Scp1. Mol Cell.

[92] Di Conza G, Trusso Cafarello S, Loroch S, Mennerich D, Deschoemaeker S, Di Matteo M (2017). The mTOR and PP2A Pathways Regulate PHD2 Phosphorylation to Fine-Tune HIF1alpha Levels and Colorectal Cancer Cell Survival under Hypoxia. Cell Rep.

[93] Yu S, Xu Z, Zou C, Wu D, Wang Y, Yao X (2014). Ion channel TRPM8 promotes hypoxic growth of prostate cancer cells via an O2 -independent and RACK1-mediated mechanism of HIF-1alpha stabilization. J Pathol.

[94] Pyo J, Ryu J, Kim W, Choi JS, Jeong JW, Kim JE (2018). The Protein Phosphatase PPM1G Destabilizes HIF-1alpha Expression. Int J Mol Sci.

[95] Takai M, Nakagawa T, Tanabe A, Terai Y, Ohmichi M, Asahi M (2015). Crosstalk between PI3K and Ras pathways via protein phosphatase 2A in human ovarian clear cell carcinoma. Cancer Biol Ther.

[96] Wen YA, Stevens PD, Gasser ML, Andrei R, Gao T (2013). Downregulation of PHLPP expression contributes to hypoxia-induced resistance to chemotherapy in colon cancer cells. Mol Cell Biol.

[97] Krtolica A, Krucher NA, Ludlow JW (1998). Hypoxia-induced pRB hypophosphorylation results from downregulation of CDK and upregulation of PP1 activities. Oncogene.

[98] Krucher NA, Rubin E, Tedesco VC, Roberts MH, Sherry TC, De Leon G (2006). Dephosphorylation of Rb (Thr-821) in response to cell stress. Exp Cell Res.

[99] Udho E, Tedesco VC, Zygmunt A, Krucher NA (2002). PNUTS (phosphatase nuclear targeting subunit) inhibits retinoblastoma-directed PP1 activity. Biochem Biophys Res Commun.

[100] Wei C, Liu X, Wang Q, Li Q, Xie M (2021). Identification of Hypoxia Signature to Assess the Tumor Immune Microenvironment and Predict Prognosis in Patients with Ovarian Cancer. Int J Endocrinol.

[101] Nyunoya T, Monick MM, Powers LS, Yarovinsky TO, Hunninghake GW (2005). Macrophages survive hyperoxia via prolonged ERK activation due to phosphatase down-regulation. J Biol Chem.

[102] Lin SP, Lee YT, Wang JY, Miller SA, Chiou SH, Hung MC (2012). Survival of cancer stem cells under hypoxia and serum depletion via decrease in PP2A activity and activation of p38-MAPKAPK2-Hsp27. PLoS One.

[103] Hofstetter CP, Burkhardt JK, Shin BJ, Gursel DB, Mubita L, Gorrepati R (2012). Protein phosphatase 2A mediates dormancy of glioblastoma multiforme-derived tumor stem-like cells during hypoxia. PLoS One.

[104] Zhou G, Golden T, Aragon IV, Honkanen RE (2004). Ser/Thr protein phosphatase 5 inactivates hypoxia-induced activation of an apoptosis signal-regulating kinase 1/MKK-4/JNK signaling cascade. J Biol Chem.

[105] Hu L, Xie H, Liu X, Potjewyd F, James LI, Wilkerson EM (2020). TBK1 Is a Synthetic Lethal Target in Cancer with VHL Loss. Cancer Discov.

[106] Zhang S, Zhou J, Shang P, Zhao G, Wang A, Mao J (2022). SET/PP2A signaling regulates macrophage positioning in hypoxic tumor regions by amplifying chemotactic responses. Exp Mol Med.

[107] Xie J, Wu D, Zhang P, Zhao S, Qi M (2024). Deciphering cutaneous melanoma prognosis through LDL metabolism: Single-cell transcriptomics analysis via 101 machine learning algorithms. Exp Dermatol.

[108] Chiu M, McBeth L, Sindhwani P, Hinds TD (2017). Deciphering the Roles of Thiazolidinediones and PPARgamma in Bladder Cancer. PPAR Res.

[109] Zhu JJ, Huang FY, Chen H, Zhang YL, Chen MH, Wu RH (2024). Autocrine phosphatase PDP2 inhibits ferroptosis by dephosphorylating ACSL4 in the Luminal A Breast Cancer. PLoS One.

[110] Wan H, Xu L, Zhang H, Wu F, Zeng W, Li T (2021). High expression of NEK2 promotes gastric cancer progression via activating AKT signaling. J Physiol Biochem.

[111] Zhou Y, Peng X, Fang C, Peng X, Tang J, Wang Z (2024). Histones Methyltransferase NSD3 Inhibits Lung Adenocarcinoma Glycolysis Through Interacting with PPP1CB to Decrease STAT3 Signaling Pathway. Adv Sci (Weinh).

[112] Rellinger EJ, Romain C, Choi S, Qiao J, Chung DH (2015). Silencing gastrin-releasing peptide receptor suppresses key regulators of aerobic glycolysis in neuroblastoma cells. Pediatr Blood Cancer.

[113] Shi Y, Wang Y, Jiang H, Sun X, Xu H, Wei X (2021). Mitochondrial dysfunction induces radioresistance in colorectal cancer by activating [Ca(2+)](m)-PDP1-PDH-histone acetylation retrograde signaling. Cell Death Dis.

[114] Karagiota A, Kanoura A, Paraskeva E, Simos G, Chachami G (2023). Pyruvate dehydrogenase phosphatase 1 (PDP1) stimulates HIF activity by supporting histone acetylation under hypoxia. FEBS J.

[115] Liu WH, Chen YJ, Chien JH, Chang LS (2014). Amsacrine suppresses matrix metalloproteinase-2 (MMP-2)/MMP-9 expression in human leukemia cells. J Cell Physiol.

[116] Wang X, Song X, Zhuo W, Fu Y, Shi H, Liang Y (2009). The regulatory mechanism of Hsp90alpha secretion and its function in tumor malignancy. Proc Natl Acad Sci U S A.

[117] Martin M, Potente M, Janssens V, Vertommen D, Twizere JC, Rider MH (2008). Protein phosphatase 2A controls the activity of histone deacetylase 7 during T cell apoptosis and angiogenesis. Proc Natl Acad Sci U S A.

[118] Jiang X, Hu J, Wu Z, Cafarello ST, Di Matteo M, Shen Y (2021). Protein Phosphatase 2A Mediates YAP Activation in Endothelial Cells Upon VEGF Stimulation and Matrix Stiffness. Front Cell Dev Biol.

[119] Ehling M, Celus W, Martin-Perez R, Alba-Rovira R, Willox S, Ponti D (2020). B55alpha/PP2A Limits Endothelial Cell Apoptosis During Vascular Remodeling: A Complementary Approach To Disrupt Pathological Vessels?. Circ Res.

[120] Minami T, Jiang S, Schadler K, Suehiro J, Osawa T, Oike Y (2013). The calcineurin-NFAT-angiopoietin-2 signaling axis in lung endothelium is critical for the establishment of lung metastases. Cell Rep.

[121] Kremer KN, Dudakovic A, McGee-Lawrence ME, Philips RL, Hess AD, Smith BD (2014). Osteoblasts protect AML cells from SDF-1-induced apoptosis. J Cell Biochem.

[122] Kremer KN, Dudakovic A, Hess AD, Smith BD, Karp JE, Kaufmann SH (2015). Histone Deacetylase Inhibitors Target the Leukemic Microenvironment by Enhancing a Nherf1-Protein Phosphatase 1alpha-TAZ Signaling Pathway in Osteoblasts. J Biol Chem.

[123] Haemmerle M, Taylor ML, Gutschner T, Pradeep S, Cho MS, Sheng J (2017). Platelets reduce anoikis and promote metastasis by activating YAP1 signaling. Nat Commun.

[124] Li T, Guo T, Liu H, Jiang H, Wang Y (2021). Platelet-derived growth factor-BB mediates pancreatic cancer malignancy via regulation of the Hippo/Yes-associated protein signaling pathway. Oncol Rep.

[125] Zhang Y, Zhen F, Sun Y, Han B, Wang H, Zhang Y (2023). Single-cell RNA sequencing reveals small extracellular vesicles derived from malignant cells that contribute to angiogenesis in human breast cancers. J Transl Med.

[126] Sellars MC, Wu CJ, Fritsch EF (2022). Cancer vaccines: Building a bridge over troubled waters. Cell.

[127] Mondal I, Das O, Sun R, Gao J, Yu B, Diaz A (2023). PP2Ac Deficiency Enhances Tumor Immunogenicity by Activating STING-Type I Interferon Signaling in Glioblastoma. Cancer Res.

[128] Raja R, Wu C, Bassoy EY, Rubino TE Jr, Utagawa EC, Magtibay PM (2022). PP4 inhibition sensitizes ovarian cancer to NK cell-mediated cytotoxicity via STAT1 activation and inflammatory signaling. J Immunother Cancer.

[129] Yang Z, Xu G, Wang B, Liu Y, Zhang L, Jing T (2021). USP12 downregulation orchestrates a protumourigenic microenvironment and enhances lung tumour resistance to PD-1 blockade. Nat Commun.

[130] Jhunjhunwala S, Hammer C, Delamarre L (2021). Antigen presentation in cancer: insights into tumour immunogenicity and immune evasion. Nat Rev Cancer.

[131] Grillone K, Riillo C, Rocca R, Ascrizzi S, Spano V, Scionti F (2022). The New Microtubule-Targeting Agent SIX2G Induces Immunogenic Cell Death in Multiple Myeloma. Int J Mol Sci.

[132] Dias MH, Liudkovska V, Montenegro Navarro J, Giebel L, Champagne J, Papagianni C (2024). The phosphatase inhibitor LB-100 creates neoantigens in colon cancer cells through perturbation of mRNA splicing. EMBO Rep.

[133] Tao M, Liu L, Shen M, Zhi Q, Gong FR, Zhou BP (2016). Inflammatory stimuli promote growth and invasion of pancreatic cancer cells through NF-kappaB pathway dependent repression of PP2Ac. Cell Cycle.

[134] Liang ZW, Ge XX, Xu MD, Qin H, Wu MY, Shen M (2021). Tumor-associated macrophages promote the metastasis and growth of non-small-cell lung cancer cells through NF-kappaB/PP2Ac-positive feedback loop. Cancer Sci.

[135] Nasri I, Bonnet D, Zwarycz B, d'Aldebert E, Khou S, Mezghani-Jarraya R (2016). PAR2-dependent activation of GSK3beta regulates the survival of colon stem/progenitor cells. Am J Physiol Gastrointest Liver Physiol.

[136] Deng M, Peng L, Li J, Liu X, Xia X, Li G (2021). PPP1R14B Is a Prognostic and Immunological Biomarker in Pan-Cancer. Front Genet.

[137] Zhuo X, Chen L, Lai Z, Liu J, Li S, Hu A (2021). Protein phosphatase 1 regulatory subunit 3G (PPP1R3G) correlates with poor prognosis and immune infiltration in lung adenocarcinoma. Bioengineered.

[138] Wang L, Dong X, Yu M, Nie X, Du M, Zhao X (2024). Association between immune-related hub genes CD36, CXCL13, FGFR4, GABBR1, LAMP3, MMP12, and PPM1H and colorectal cancer prognosis. Am J Transl Res.

[139] Yu Z, Song Y, Cai M, Jiang B, Zhang Z, Wang L (2021). PPM1D is a potential prognostic biomarker and correlates with immune cell infiltration in hepatocellular carcinoma. Aging (Albany NY).

[140] Chen X, Wang Y, Li Y, Liu G, Liao K, Song F (2022). Identification of immune-related cells and genes in the breast invasive carcinoma microenvironment. Aging (Albany NY).

[141] Metcalfe S, Milner J (1990). Phosphatases PP1 and PP2A act after the G0/G1 interface in lymphocyte activation. Immunol Lett.

[142] Wang F, Zhang Y, Yu X, Teng XL, Ding R, Hu Z (2021). ZFP91 disturbs metabolic fitness and antitumor activity of tumor-infiltrating T cells. J Clin Invest.

[143] Yan Q, Zhang B, Ling X, Zhu B, Mei S, Yang H (2022). CTLA-4 Facilitates DNA Damage-Induced Apoptosis by Interacting With PP2A. Front Cell Dev Biol.

[144] Eil R, Vodnala SK, Clever D, Klebanoff CA, Sukumar M, Pan JH (2016). Ionic immune suppression within the tumour microenvironment limits T cell effector function. Nature.

[145] Medyouf H, Alcalde H, Berthier C, Guillemin MC, dos Santos NR, Janin A (2007). Targeting calcineurin activation as a therapeutic strategy for T-cell acute lymphoblastic leukemia. Nat Med.

[146] Shui JW, Hu MC, Tan TH (2007). Conditional knockout mice reveal an essential role of protein phosphatase 4 in thymocyte development and pre-T-cell receptor signaling. Mol Cell Biol.

[147] Brechmann M, Mock T, Nickles D, Kiessling M, Weit N, Breuer R (2012). A PP4 holoenzyme balances physiological and oncogenic nuclear factor-kappa B signaling in T lymphocytes. Immunity.

[148] Mourtada-Maarabouni M, Williams GT (2009). Protein phosphatase 4 regulates apoptosis in leukemic and primary human T-cells. Leuk Res.

[149] Apostolidis SA, Rodriguez-Rodriguez N, Suarez-Fueyo A, Dioufa N, Ozcan E, Crispin JC (2016). Phosphatase PP2A is requisite for the function of regulatory T cells. Nat Immunol.

[150] He S, Li J, Cheng P, Zeng Z, Zhang C, Duan H (2021). Charge-Reversal Polymer Nano-modulators for Photodynamic Immunotherapy of Cancer. Angew Chem Int Ed Engl.

[151] Ghione S, Racoeur C, Mabrouk N, Shan J, Groetz E, Ballot E (2022). Protein Kinase Inhibitor-Mediated Immunoprophylactic and Immunotherapeutic Control of Colon Cancer. Front Immunol.

[152] Cui J, Wang H, Medina R, Zhang Q, Xu C, Indig IH (2020). Inhibition of PP2A with LB-100 Enhances Efficacy of CAR-T Cell Therapy Against Glioblastoma. Cancers (Basel).

[153] Meidan E, Li H, Pan W, Kono M, Yu S, Kyttaris VC (2020). Serine/threonine phosphatase PP2A is essential for optimal B cell function. JCI Insight.

[154] Zeng Q, Zhang H, Qin J, Xu Z, Gui L, Liu B (2015). Rapamycin inhibits BAFF-stimulated cell proliferation and survival by suppressing mTOR-mediated PP2A-Erk1/2 signaling pathway in normal and neoplastic B-lymphoid cells. Cell Mol Life Sci.

[155] Sebestyen A, Hajdu M, Kis L, Barna G, Kopper L (2007). Smad4-independent, PP2A-dependent apoptotic effect of exogenous transforming growth factor beta 1 in lymphoma cells. Exp Cell Res.

[156] Brandsma AM, Schwartz SL, Wester MJ, Valley CC, Blezer GLA, Vidarsson G (2018). Mechanisms of inside-out signaling of the high-affinity IgG receptor FcgammaRI. Sci Signal.

[157] Gu M, Zhang T, lin W, Liu Z, Lai R, Xia D (2014). Protein phosphatase PP1 negatively regulates the Toll-like receptor- and RIG-I-like receptor-triggered production of type I interferon by inhibiting IRF3 phosphorylation at serines 396 and 385 in macrophage. Cell Signal.

[158] Liang Y, Sun X, Wang M, Lu Q, Gu M, Zhou L (2021). PP2Acalpha promotes macrophage accumulation and activation to exacerbate tubular cell death and kidney fibrosis through activating Rap1 and TNFalpha production. Cell Death Differ.

[159] Long L, Deng Y, Yao F, Guan D, Feng Y, Jiang H (2014). Recruitment of phosphatase PP2A by RACK1 adaptor protein deactivates transcription factor IRF3 and limits type I interferon signaling. Immunity.

[160] Ho WS, Mondal I, Xu B, Das O, Sun R, Chiou P (2023). PP2Ac/STRN4 negatively regulates STING-type I IFN signaling in tumor-associated macrophages. J Clin Invest.

[161] Qian Y, Yuan J, Hu H, Yang Q, Li J, Zhang S (2015). The CUL4B/AKT/beta-Catenin Axis Restricts the Accumulation of Myeloid-Derived Suppressor Cells to Prohibit the Establishment of a Tumor-Permissive Microenvironment. Cancer Res.

[162] Perego J, Mendes A, Bourbon C, Camosseto V, Combes A, Liu H (2018). Guanabenz inhibits TLR9 signaling through a pathway that is independent of eIF2alpha dephosphorylation by the GADD34/PP1c complex. Sci Signal.

[163] Wang Y, Yang H, Jia A, Wang Y, Yang Q, Dong Y (2022). Dendritic cell Piezo1 directs the differentiation of T(H)1 and T(reg) cells in cancer. Elife.

[164] Al Johani KA, Hegarty AM, Porter SR, Fedele S (2009). Calcineurin inhibitors in oral medicine. J Am Acad Dermatol.

[165] Salowe SP, Hermes JD (1998). Competitive and slow-binding inhibition of calcineurin by drug x immunophilin complexes. Arch Biochem Biophys.

[166] Grassberger M, Baumruker T, Enz A, Hiestand P, Hultsch T, Kalthoff F (1999). A novel anti-inflammatory drug, SDZ ASM 981, for the treatment of skin diseases: *in vitro* pharmacology. Br J Dermatol.

[167] Gardner LA, Klawitter J, Gregory MA, Zaberezhnyy V, Baturin D, Pollyea DA (2014). Inhibition of calcineurin combined with dasatinib has direct and indirect anti-leukemia effects against BCR-ABL1(+) leukemia. Am J Hematol.

[168] Dotto GP (2011). Calcineurin signaling as a negative determinant of keratinocyte cancer stem cell potential and carcinogenesis. Cancer Res.

[169] Pagano MA, Tibaldi E, Molino P, Frezzato F, Trimarco V, Facco M (2019). Mitochondrial apoptosis is induced by Alkoxy phenyl-1-propanone derivatives through PP2A-mediated dephosphorylation of Bad and Foxo3A in CLL. Leukemia.

[170] Vicente C, Arriazu E, Martinez-Balsalobre E, Peris I, Marcotegui N, Garcia-Ramirez P (2020). A novel FTY720 analogue targets SET-PP2A interaction and inhibits growth of acute myeloid leukemia cells without inducing cardiac toxicity. Cancer Lett.

[171] Leonard D, Huang W, Izadmehr S, O'Connor CM, Wiredja DD, Wang Z (2020). Selective PP2A Enhancement through Biased Heterotrimer Stabilization. Cell.

[172] Kauko O, O'Connor CM, Kulesskiy E, Sangodkar J, Aakula A, Izadmehr S (2018). PP2A inhibition is a druggable MEK inhibitor resistance mechanism in KRAS-mutant lung cancer cells. Sci Transl Med.

[173] Jayappa KD, Tran B, Gordon VL, Morris C, Saha S, Farrington CC (2023). PP2A modulation overcomes multidrug resistance in chronic lymphocytic leukemia via mPTP-dependent apoptosis. J Clin Invest.

[174] De Falco F, Rompietti C, Sorcini D, Esposito A, Scialdone A, Baldoni S (2022). GSK3beta is a critical, druggable component of the network regulating the active NOTCH1 protein and cell viability in CLL. Cell Death Dis.

[175] Chung V, Mansfield AS, Braiteh F, Richards D, Durivage H, Ungerleider RS (2017). Safety, Tolerability, and Preliminary Activity of LB-100, an Inhibitor of Protein Phosphatase 2A, in Patients with Relapsed Solid Tumors: An Open-Label, Dose Escalation, First-in-Human, Phase I Trial. Clin Cancer Res.

[176] Ho WS, Sizdahkhani S, Hao S, Song H, Seldomridge A, Tandle A (2018). LB-100, a novel Protein Phosphatase 2A (PP2A) inhibitor, sensitizes malignant meningioma cells to the therapeutic effects of radiation. Cancer Lett.

[177] Ronk H, Rosenblum JS, Kung T, Zhuang Z (2022). Targeting PP2A for cancer therapeutic modulation. Cancer Biol Med.

[178] Tan DS, Lambros MB, Rayter S, Natrajan R, Vatcheva R, Gao Q (2009). PPM1D is a potential therapeutic target in ovarian clear cell carcinomas. Clin Cancer Res.

[179] Buss MC, Read TA, Schniederjan MJ, Gandhi K, Castellino RC (2012). HDM2 promotes WIP1-mediated medulloblastoma growth. Neuro Oncol.

[180] Cao X, Dang L, Zheng X, Lu Y, Lu Y, Ji R (2019). Targeting Super-Enhancer-Driven Oncogenic Transcription by CDK7 Inhibition in Anaplastic Thyroid Carcinoma. Thyroid.

[181] Xiong S, Zhou J, Tan TK, Chung TH, Tan TZ, Toh SH (2024). Super enhancer acquisition drives expression of oncogenic PPP1R15B that regulates protein homeostasis in multiple myeloma. Nat Commun.

[182] Winkler C, De Munter S, Van Dessel N, Lesage B, Heroes E, Boens S (2015). The selective inhibition of protein phosphatase-1 results in mitotic catastrophe and impaired tumor growth. J Cell Sci.

[183] Martin-Granados C, Prescott AR, Van Dessel N, Van Eynde A, Arocena M, Klaska IP (2012). A role for PP1/NIPP1 in steering migration of human cancer cells. PLoS One.

[184] Lai X, Lui SKL, Lam HY, Adachi Y, Sim WJ, Vasilevski N (2023). SHP2 inhibitors maintain TGFbeta signalling through SMURF2 inhibition. NPJ Precis Oncol.

[185] Xie W, Sun Y, Zeng Y, Hu L, Zhi J, Ling H (2022). Comprehensive analysis of PPPCs family reveals the clinical significance of PPP1CA and PPP4C in breast cancer. Bioengineered.

[186] Liu Y, Wang Y, Sun S, Chen Z, Xiang S, Ding Z (2022). Understanding the versatile roles and applications of EpCAM in cancers: from bench to bedside. Exp Hematol Oncol.

[187] Wang L, Lin Y, Yao Z, Babu N, Lin W, Chen C (2024). Targeting undruggable phosphatase overcomes trastuzumab resistance by inhibiting multi-oncogenic kinases. Drug Resist Updat.

[188] Kolupaeva V, Janssens V (2013). PP1 and PP2A phosphatases-cooperating partners in modulating retinoblastoma protein activation. FEBS J.

[189] Van Itallie CM, Aponte A, Tietgens AJ, Gucek M, Fredriksson K, Anderson JM (2013). The N and C termini of ZO-1 are surrounded by distinct proteins and functional protein networks. J Biol Chem.

[190] Yu YM, Pace SM, Allen SR, Deng CX, Hsu LC (2008). A PP1-binding motif present in BRCA1 plays a role in its DNA repair function. Int J Biol Sci.

[191] Novoa I, Zeng H, Harding HP, Ron D (2001). Feedback inhibition of the unfolded protein response by GADD34-mediated dephosphorylation of eIF2alpha. J Cell Biol.

[192] Paul D, Bargale AB, Rapole S, Shetty PK, Santra MK (2019). Protein Phosphatase 1 Regulatory Subunit SDS22 Inhibits Breast Cancer Cell Tumorigenesis by Functioning as a Negative Regulator of the AKT Signaling Pathway. Neoplasia.

[193] Mannava S, Omilian AR, Wawrzyniak JA, Fink EE, Zhuang D, Miecznikowski JC (2012). PP2A-B56alpha controls oncogene-induced senescence in normal and tumor human melanocytic cells. Oncogene.

[194] Elgenaidi IS, Spiers JP (2019). Regulation of the phosphoprotein phosphatase 2A system and its modulation during oxidative stress: A potential therapeutic target?. Pharmacol Ther.

[195] Liu CC, Lin SP, Hsu HS, Yang SH, Lin CH, Yang MH (2016). Suspension survival mediated by PP2A-STAT3-Col XVII determines tumour initiation and metastasis in cancer stem cells. Nat Commun.

[196] Mammucari C, Tommasi di Vignano A, Sharov AA, Neilson J, Havrda MC, Roop DR (2005). Integration of Notch 1 and calcineurin/NFAT signaling pathways in keratinocyte growth and differentiation control. Dev Cell.

[197] Ryeom S, Baek KH, Rioth MJ, Lynch RC, Zaslavsky A, Birsner A (2008). Targeted deletion of the calcineurin inhibitor DSCR1 suppresses tumor growth. Cancer Cell.

